# Microglia in Alzheimer Disease: Well-Known Targets and New Opportunities

**DOI:** 10.3389/fnagi.2019.00233

**Published:** 2019-08-30

**Authors:** Anne-Laure Hemonnot, Jennifer Hua, Lauriane Ulmann, Hélène Hirbec

**Affiliations:** Institute for Functional Genomics (IGF), University of Montpellier, Centre National de la Recherche Scientififique, Institut National de la Santé et de la Recherche Médicale, Montpellier, France

**Keywords:** Alzheimer disease, microglia, neuroinflammation, microglia diversity, purinergic signaling, sexual dimorphism, early stage, hiPSCs

## Abstract

Microglia are the resident macrophages of the central nervous system. They play key roles in brain development, and physiology during life and aging. Equipped with a variety of molecular sensors and through the various functions they can fulfill, they are critically involved in maintaining the brain’s homeostasis. In Alzheimer disease (AD), microglia reaction was initially thought to be incidental and triggered by amyloid deposits and dystrophic neurites. However, recent genome-wide association studies have established that the majority of AD risk loci are found in or near genes that are highly and sometimes uniquely expressed in microglia. This leads to the concept of microglia being critically involved in the early steps of the disease and identified them as important potential therapeutic targets. Whether microglia reaction is beneficial, detrimental or both to AD progression is still unclear and the subject of intense debate. In this review, we are presenting a state-of-knowledge report intended to highlight the variety of microglial functions and pathways shown to be critically involved in AD progression. We first address both the acquisition of new functions and the alteration of their homeostatic roles by reactive microglia. Second, we propose a summary of new important parameters currently emerging in the field that need to be considered to identify relevant microglial targets. Finally, we discuss the many obstacles in designing efficient therapeutic strategies for AD and present innovative technologies that may foster our understanding of microglia roles in the pathology. Ultimately, this work aims to fly over various microglial functions to make a general and reliable report of the current knowledge regarding microglia’s involvement in AD and of the new research opportunities in the field.

## Introduction

Microglia cells are the main immunocompetent cells in the brain. They colonize the brain in the early prenatal period (Ginhoux et al., 2010), but contrary to other tissue resident macrophages, they remain secluded within the CNS throughout life and self-renew at slow pace (Ajami et al., 2007). Importantly, the CNS microenvironment significantly shapes the microglia’s phenotype, endowing them with specific important homeostatic and supportive brain functions (Kierdorf and Prinz, 2017). Should the brain homeostasis be compromised, microglia change their phenotype and initiate a defense program. Thus, under pathological conditions, they adopt reactive states characterized by multiple morphological and functional changes including but not limited to increased phagocytosis and increased expression of receptors, cytokines, chemokines and additional inflammation related molecules (Wolf et al., 2017).

Alzheimer’s disease (AD) classical hallmarks include brain atrophy, extracellular amyloid-beta (Aβ) deposits, intracellular aggregated phosphorylated tau, dystrophic neurites, synapses and neurons loss (Bedner et al., 2015). The presence of reactive glial cells within the neuritic plaques was described by Alois Alzheimer himself (Alzheimer, 1907; Graeber et al., 1997) and further studies identified both reactive astrocytes and microglia in the vicinity of the Aβ deposits (Verkhratsky et al., 2016). Long considered as a consequence of the pathology, reactive glia and associated neuroinflammation are now regarded as playing key roles in both disease initiation and progression. Indeed, Human genetic studies identified over 25 genetic loci that robustly associate with AD risk (Hansen et al., 2018; Verheijen and Sleegers, 2018). Among them, most of the common (ApoE, Sp1l) or rare (Trem2, Cd33) genetic variants code for proteins that are preferentially or exclusively expressed in microglia. These findings strongly support a causal involvement of microglial cells in AD pathogenesis and generated a strong interest for studying these cells in AD. Yet, the roles of microglia in AD initiation and progression are unclear and heavily debated, with conflicting reports regarding their detrimental or protective contribution to the disease.

In the present review, we have summarized the main findings regarding the role of microglia in AD. Microglia reaction is known to be associated with the acquisition of many immune functions which are triggered by the activation of receptors designed to recognize danger or pathogen associated molecular patterns (DAMPs/PAMPs). Its role is to restore homeostasis and is also associated with the loss or the alteration of homeostatic functions which are important for brain physiological functioning. In the two first parts, we thus provide an overview of the microglial functions and pathways that are known to be altered during AD. We then highlight factors such as mouse models, sex, age whose influence may have been under-examined in assessing the contribution of microglial cells to the disease progression. Finally, we identified new research topics that are likely to foster our understanding of the roles of microglia to AD initiation and progression and may help design more targeted therapeutic strategies.

## New Functions for Reactive Microglia in AD

**FIGURE 1:**
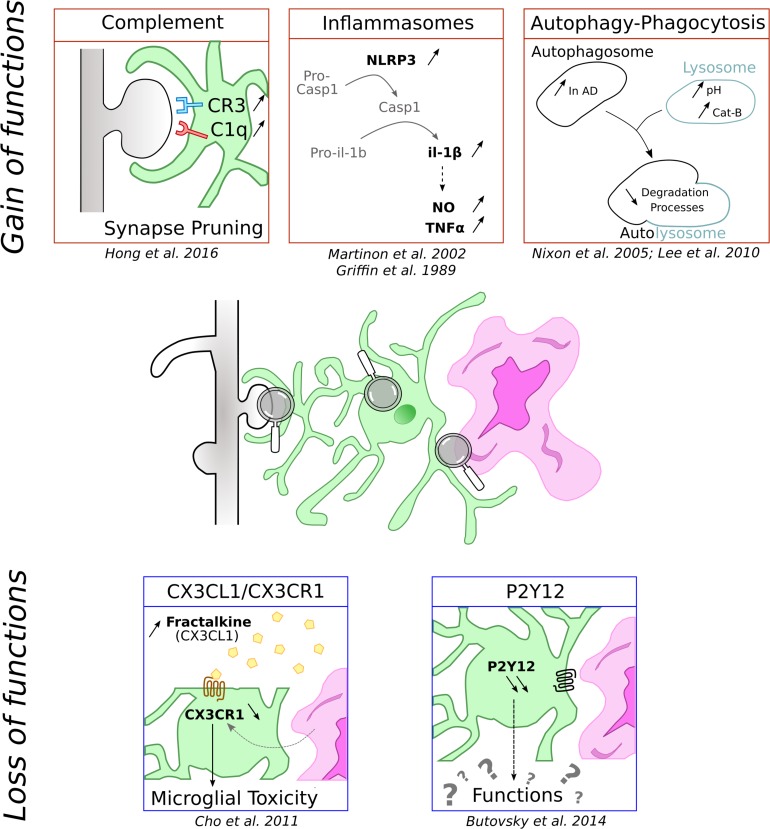
Schematic representation of functions microglial cells can loose and gain in context of AD. Microglia is represented in green associated to amyloid-β deposit in purple and dendritic spines in gray.

Neuroinflammation is a common feature of neurodegenerative diseases and inflammatory processes are thus among the most studied microglial functions in AD. Microglia, which represent the main immune cells of the brain, have been shown to play key roles in orchestrating this brain inflammation. In the following section, we are reporting the main microglial processes involved in neuroinflammation (Figure 1, top part). However, more detailed description of these processes can be found in recent reviews that are focusing on these specific points (Labzin et al., 2017; Nizami et al., 2019).

### Inflammasomes Are Central Hubs for Cytokines Production

One of inflammation hallmarks is the release of cytokines. This secretion requires the activation of inducible multiproteic complexes called inflammasomes. Several inflammasomes have been characterized but the most important microglial contributor in pathologies is certainly the NLRP3. It is composed of the sensor protein NLRP3 and the adaptor protein apoptosis associated Speck-like protein (ASC), which contains a caspase recruitment domain. ASC can recruit and activate pro-caspase-1. When stimulated, the complex induces the cleavage of pro-caspase-1 into active caspase-1, which in turn cleaves pro-IL-1β and IL-18 triggering their release in the extracellular space (Martinon et al., 2002). NLRP3 activation pathway is not fully characterized, but the current view is that NLRP3 activation requires the occurrence of two independent but co-concomitant priming and activation signals (Próchnicki et al., 2016).

In the context of AD, IL-1β is known to elicit the secretion of NO and TNFα, promoting the formation of deleterious amyloid plaques and neuronal degeneration (Griffin et al., 1989). In accordance with these data, frontal cortex from AD patients exhibit an increase of caspase-1 which is correlated with an attenuation of Aβ peptide phagocytosis (Burguillos et al., 2011; Heneka et al., 2013). Likewise, genetic deletion of NLRP3 in mice with familiar AD associated mutation reduces the level of IL-1β and Aβ deposits, and correlates with positive impacts on synaptic dysfunction and cognitive performances (Heneka et al., 2013).

Other members of the caspase family are also involved in AD pathology. Interestingly, the activity of caspase-3 prematurely increases in hippocampal neurons in an AD mouse model (D’Amelio et al., 2011). Similarly, in samples from AD patients, caspase-8 and -3 are upregulated in cortical microglia (Burguillos et al., 2011). Microglial caspase-3 around amyloid plaques also present a cytosolic location, suggesting a non-apoptotic role of caspases in AD (Burguillos et al., 2011). Such evidence has also been described in others degenerative models such as brain ischemia, in which reactive astrocytes and microglia express cytoplasmic non-apoptotic caspase-3 (Wagner et al., 2011). This non-nuclear localization could be involved in cytoplasmic rearrangement and modifications of cell populations surrounding lesions. Although it is undeniable that caspase signaling is crucial in the development of AD, the delay of activation and the pathway downstream of each of them need to be clarified.

### The Complement System, the Microglial Way of Eating Synapses?

The complement pathway is part of the innate immune system and mediates the recognition and elimination of pathogens and cellular debris. It is involved in many physiological and pathological functions throughout life, including activity-dependent synapse elimination during development (Schafer et al., 2012). In AD, reactivation of this pathway by Aβ deposits has been associated with synapse loss.

The complement cascade is composed of a large panel of mediators including C1q and C3 complex proteins which can be activated by three different pathways, all of which are capable of triggering phagocytosis (Stephan et al., 2012). In the CNS, complement proteins are expressed in neurons and glial cells, although microglia and astrocytes are the major sources of complement. Particularly, microglia expressed high level of C1q and CR3 (Veerhuis et al., 2011) and microglial CR3 have been shown to be crucial in synapse pruning during development (Schafer et al., 2012).

In AD context, patients showed elevated CSF concentrations of C3 and CR1, pointing out an alteration of complement system in the pathology (Daborg et al., 2012). The complement has been associated with Aβ but it is not clear whether it is protective or detrimental. Indeed, some studies reported that complement inhibition or deficiency results in accelerated amyloid pathology (Wyss-Coray et al., 2002; Maier et al., 2008) and that C3 along with CR3 contribute to Aβ phagocytosis (Fu et al., 2012). On the other hand, other studies pointed out that elimination and modulation of microglial CR3 decrease Aβ level (Czirr et al., 2017) and C3 antagonist ameliorates plaque load (Lian et al., 2016). Further studies are thus required to better understand the role of complement in Aβ pathology.

As AD is marked by an important synapse loss, questions have been raised on whether the complement could mediate such synapse elimination. Fonseca et al. (2004) demonstrated that C1q deficiency in an AD mouse model partly restores synapse integrity pointing out a role of the complement system in AD. More recently, works from Hong et al. demonstrated that microglial C3/CR3 mediates synapse elimination when challenged with oligomeric Aβ (Hong et al., 2016). More specifically, they found that C1q is upregulated into synapse early in J20 mice and that Aβ oligomers increase C1q and microglia phagocytic activity. This resulted in synapse elimination by microglia, a process which is lost in CR3 KO mice. This study proposes a model in which C1q and oligomer Aβ would activate the complement cascade to drive synapse elimination through microglial CR3.

### Eat Me and Eat Myself

Autophagy and phagocytosis are cellular degradation processes, necessary to degrade additional or damaged particles in lysosomes. These processes, ensured by a large enzymatic degradation system, are dysregulated during aging and are of particular importance during AD, as shown with the autophagy failure and the increase of autophagosomes in AD patients (Nixon et al., 2005). Moreover, lysosomal acidification and autophagy are disrupted by Alzheimer-related PS1 mutation (Lee et al., 2010). Numerous studies demonstrate that microglial Aβ phagocytosis contribute to degeneration by triggering NLRP3 and lysosomal cathepsin-B that subsequently results in maturation and release of IL-1β (Halle et al., 2008). Cellular degradation processes could thus, by differently modulating the inflammasome, present opposite effects. It could be protective in normal physiological states and during the premature state of the pathology, and detrimental during chronic and late phases of diseases.

## Loss of Homeostatic Functions in Reactive Microglia

Although the majority of the studies concentrate on the microglial reactivity-acquired functions and assess their contribution to neurodegeneration, loss of key homeostatic functions may also be detrimental to neuronal functions and may contribute to the detrimental effects of microglia reaction. In the following part, we are reviewing few key microglial functions that are compromised in AD (Figure 1, bottom part).

### Consequences of CX3CL1/CX3CR1 Signaling Loss in AD

In brain, the CX3CR1 receptor is predominantly expressed in microglia. Its ligand is the secreted soluble form of fractalkine, also named CX3CL1, and is constitutively expressed by neurons. CX3CL1 exerts an inhibitory signal, maintaining microglia in a resting state. CX3CL1-CX3CR1 is a critical signaling pathway during development as shown by the delay of glutamatergic synapse maturation (Paolicelli et al., 2011; Hoshiko et al., 2012) and functional consequences in adult synapses (Basilico et al., 2019) in CX3CR1^–/–^ mice. Age is also a decisive factor in the regulation of CX3CR1 expression as LPS challenge is responsible for a more pronounced impairment of CX3CR1 expression in aged compared to young rats (Lyons et al., 2009).

The roles of CX3CL1/CX3CR1 communication during neuroinflammation are still subject to debate since CX3CR1 deletion effects differ depending on the challenge. In CX3CR1^–/–^ mice and in both PD and ALS models, Cardona et al. (2006) demonstrated an extensive neuronal loss due to an alteration of cytokines production. CX3CR1 decrease is also observed in AD models. In neurodegenerative conditions, this disruption is associated with a strong microglial toxicity and an aggravation of the pathology (Keren-Shaul et al., 2017). The involvement of the CX3CL1/CX3CR1 signaling pathway in AD is confirmed by an increase in the plasmatic concentration of CX3CL1 in AD and MCI patients compared to healthy control subjects (Kim et al., 2008). However, the role of this pathway might be more complex as CX3CR1 deletion was shown to prevent neuronal loss in 3 × Tg AD mice (Fuhrmann et al., 2010) but worsen cellular and behavioral deficits in hAPP-J20 mice (Cho et al., 2011).

All these data point out to critical roles for CX3CL1–CX3CR1 signaling during neurodegenerative diseases, including AD, but also demonstrate that its complex spectrum of responses may depend on the genetic model of the disease.

### The Yet Unresolved Role of P2Y12 Down-Regulation in AD

In physiological conditions, P2Y12 receptor is involved in chemotaxis and mice lacking this receptor showed altered microglia migration and polarization (Haynes et al., 2006). *P2ry12* was identified as a unique microglia gene in the CNS (Butovsky et al., 2014). It is one of the most highly expressed genes in microglia and is downregulated in reactive microglia (Haynes et al., 2006). Consequently, *P2ry12* gene expression levels have been proposed to be a marker of the homeostatic microglial signature (Butovsky et al., 2014). In agreement, in AD transgenic mouse model, microglia located at proximity of amyloid plaques do not express P2Y12R whereas the receptor is observed in plaque-distant ones (Butovsky et al., 2014; Jay et al., 2015). Similar findings have also been reported in human AD patients (Sanchez-Mejias et al., 2016; Mildner et al., 2017). However, so far, the consequences of this down-regulation for microglia functions are unknown and merit further attention.

## Most Studied Microglial Molecular Targets in AD

In the past years, genome-wide association studies (GWAS) identified over 25 genetic loci that robustly associate with risk of late onset Alzheimer disease (LOAD); many of these, relate to neuroinflammation and are preferentially or exclusively expressed in microglial cells, implicating microglia reaction as not only a consequence of Alzheimer’s but likely also a cause. In this section, we are reviewing the current knowledge regarding the roles of the most studied genes in this context.

### APOE: Beyond Aβ Modulation, a Microglia-Function Modifier

The ε4 isoform of the Apolipoprotein E (APOE) represents a common genetic variant associated with AD and is the most significant known risk factor. APOE is an apolipoprotein implicated in cholesterol and lipid transfer between cells. In the brain, it is produced mainly by astrocytes, but also by microglia and to a lesser extent by neurons. In humans, APOE is found in three main isoforms: ε2, ε3, and ε4. The APOE-ε4 isoform represents the most significant risk factor for LOAD: the presence of one APOE-ε4 copy increases the risk of developing LOAD by three-fold whereas two APOE-ε4 copies lead to a nine-fold increase (Corder et al., 1993). On the opposite, individuals carrying the rare ε2 variant are less likely to develop AD and the most common APOE-ε3 isoform is thought to be neutral (Serrano-Pozo et al., 2015). How APOE isoforms affect the onset and development of AD remains unclear. Based on the early-described interaction between APOE and Aβ, studies mainly focused on the effects of APOE on amyloid load and oligomerization, which was shown to be isoforms-dependent (Strittmatter et al., 1993; Naslund et al., 1995; Hashimoto et al., 2012). Thus, APOE-ε4 patients have more Aβ plaques and oligomers than ε3 carriers (Schmechel et al., 1993; Tiraboschi et al., 2004; Hashimoto et al., 2012; Koffie et al., 2012), and mouse models expressing human APOE isoforms mimics the isoform-dependent modifications on Aβ deposits (Fagan et al., 2002; Hudry et al., 2013; Zhao et al., 2014). It was also reported that APOE can also influence Aβ clearance (DeMattos et al., 2004; Castellano et al., 2011), however, APOE-knockdown mice develop less Aβ deposits (Holtzman et al., 1999; Fagan et al., 2002).

Little is known regarding the microglial role of APOE. Early post-mortem studies in humans found a higher number of reactive microglia in APOE-ε4 carriers compared to APOE-ε3 (Egensperger et al., 1998), and recently Minett et al. (2016) found that APOE-ε4 allele was strongly associated with reactive microglia. Studies using mouse models expressing human isoforms also showed increased microgliosis in APOE-ε4 expressing mice compared to APOE-ε3 (Belinson and Michaelson, 2009; Rodriguez et al., 2014). Overall, these studies highlight a relation between APOE isoforms and reactive microglia. Evidences have also been pointing out a role of APOE in inflammatory processes (Lynch et al., 2001; Thangavel et al., 2017). Thus, APOE is up-regulated in disease-associated microglia (DAMs) and modulates transcription of homeostatic and inflammatory factors (Krasemann et al., 2017). Moreover, APOE modifies inflammatory response in an isoform manner; APOE-ε4 expressing mice releasing more pro-inflammatory cytokines than APOE-ε3 (Guo et al., 2004; Vitek et al., 2009; Zhu et al., 2012). However, the concept of APOE triggering more pro-inflammatory response has been challenged since APOE in the presence of Aβ can induce a decrease in pro-inflammatory cytokine release (Guo et al., 2004), indicating a more complex link between APOE, Aβ and glial cells. Overall, APOE is undoubtedly implicated in LOAD through Aβ aggregation and clearance but also by modulating microglia activation and cytokine release. However, additional studies are needed to explore these processes.

### TREM2, the Well-Known Risk Factor With Unclarified Roles

Aside from APOE, the other major well-studied gene associated with LOAD is the Triggering receptor expressed on myeloid cells 2 (TREM2). Indeed, GWAS studies identified several rare Trem2 gene variants associated with LOAD (Guerreiro et al., 2013; Jin et al., 2014). Among them, the rs75932628 variant, which causes the loss-of-function R47H mutation, showed significant association with AD and suggests a protective role for TREM2 activation pathway in AD (Guerreiro et al., 2013; Jonsson et al., 2013). TREM2 is a cell surface receptor expressed in myeloid cells, including microglia (Schmid et al., 2002; Kiialainen et al., 2005) and was shown to modulate inflammation (Piccio et al., 2007), phagocytosis (Takahashi et al., 2005) and chemokine secretion (Bouchon et al., 2002; Sieber et al., 2013). How TREM2 affect AD is currently not well understood but TREM2 is increased in APP/PS1 hippocampus and cortices (Jiang et al., 2014) and its expression increases with age (Jay et al., 2015). Moreover, TREM2 expression is increased in plaque-associated microglia (Frank et al., 2008; Guerreiro et al., 2013), and modulating TREM2 expression can reprogram microglia response (Krasemann et al., 2017; Lee C.Y.D. et al., 2018). In particular, Keren-Shaul et al. demonstrated that TREM2 is required for a complete activation of DAMs (Keren-Shaul et al., 2017), pointing out a major role of TREM2 in those cells.

Most of the studies on TREM2 in AD relate to microglia-mediated Aβ phagocytosis, however, they do not all agree on whether it has beneficial or detrimental effects. Indeed, Ulrich et al. (2014) found no change in amyloid burden in 3-month-old TREM2-heterozygous APP-21 mice whereas Jay et al. (2015) showed reduced 6E10 staining in 4-month-old TREM2 deficient APP/PS1 mice. On the opposite, Wang et al. (2015) suggested using 8-month-old 5xFAD mice, that TREM2 deficiency would be detrimental as it increases hippocampal Aβ peptide. Similarly, Jiang et al. (2014) showed, *in vitro*, that TREM2 facilitates Aβ 1-42 phagocytosis and, *in vivo*, that TREM2 overexpression reduces plaque density in the cortex and hippocampus of APP/PS1 mice. Altogether, these different studies suggest a complex role of TREM2 on Aβ and suggest an age- or stage-dependent effect.

Contradictory results have also been found in TREM2-dependent inflammatory response. Jay et al. (2015) showed that TREM2 deficiency in APP/PS1 mice reduces the pro-inflammatory response. On the contrary, in *in vitro* studies, TREM2 overexpression was shown to reduce - while TREM2 deficiency increases - pro-inflammatory cytokine production (Takahashi et al., 2005; Jiang et al., 2014). Therefore, further studies are required to clarify how TREM2 influences inflammatory response.

Despite discrepancies found in TREM2 implication in inflammatory response and Aβ deposition, it is of agreement that TREM2 deficiency causes a decrease in Aβ-associated microglia (Jay et al., 2015; Wang et al., 2015; Zhao et al., 2018). Altogether, those studies show TREM2 implication in AD by modulating inflammatory processes and Aβ deposition. Moreover, several studies found that Aβ binds to TREM2 and that this interaction can modulate microglia functions such as proliferation, Aβ degradation and inflammatory response (Zhong et al., 2018). Interestingly, a link between TREM2 and APOE has recently been highlighted suggesting that TREM2 modulates APOE expression and signaling (Krasemann et al., 2017; Parhizkar et al., 2019).

Many studies aim to decipher the role of TREM2 in AD. However, current studies mainly used TREM2 overexpression or knockout although it might be relevant to also study TREM2 variants.

### Other AD Risk Factors

Alzheimer disease-related GWAS have identified many other microglia genes as potential risk factors in AD. This includes genes such as *Cd33*, *Cr1*, or *Abca7*, which have been implicated in phagocytosis (Villegas-Llerena et al., 2016). Additionally, CD33 was shown to inhibit Aβ clearance whereas CR1 is part of the complement pathway that helps eliminating Aβ plaques. Various SNPs of CD33 have been found: the rs3826656 and rs3865444 variants are associated with LOAD whereas the rs3865444 variant confers protection (McQuade and Blurton-Jones, 2019). Overall, more than 25 microglia genes have been highlighted by GWAS studies (Verheijen and Sleegers, 2018). Their contribution to AD pathogenesis is yet unknown and merit scientists’ attention as they tend to be more common genetic variants.

## Emerging Microglial Targets in AD

**FIGURE 2:**
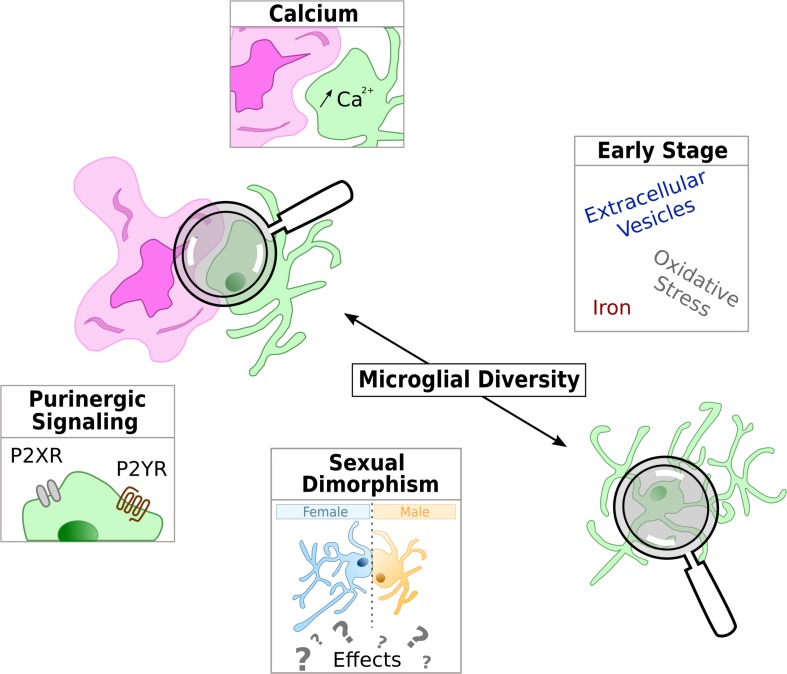
Schematic representation of the current hot topics and emerging microglial targets in AD physiopathology studied in this review. Microglia is represented in green and either with a reactive shape associated to amyloid-β deposit in purple or in the parenchyma with an homeostatic shape, away from Aβ.

Emerging microglial targets are highlighted in Figure 2.

### Purinergic Signaling

Of the many mechanisms implicated in microglial functions, purinergic signaling is one worth mentioning. Indeed, microglial purinergic receptors are known to modulate several processes including phagocytosis, chemotaxis and cytokine release (Abbracchio and Ceruti, 2007; Calovi et al., 2019). Purinergic receptors are divided into two super families: P1 receptors respond to adenosine whereas P2 respond to ADP and ATP. Purinergic receptors are widely expressed in several CNS cell types including microglia (Butovsky et al., 2014), and both ionotropic P2X and metabotropic P2Y receptors have been implicated in neurological diseases including AD (Burnstock, 2016).

Of the G protein-coupled P2Y receptors, P2Y6R, 12, and 13 are the three most investigated subtypes expressed in microglia (Calovi et al., 2019). In AD, the most studied is P2Y12 receptor which plays important role in homeostatic microglia and whose role is described above (see section “Loss of Homeostatic Functions in Reactive Microglia”). As for the other P2YRs, P2Y6R might be implicated in amyloid phagocytosis as Wendt et al. (2017) demonstrate an impaired P2Y6R-dependent phagocytosis mechanism in 9-month-old 5xFAD mice. Although P2Y2R expression in microglia seems very low (Calovi et al., 2019), studies have also investigate its implication in AD. *In vitro*, Aβ treatment of primary microglia induces an increase of P2Y2R expression and Aβ-treated P2Y2R^–/–^ microglia showed loss of motility and altered ATP/UDP-triggered Aβ uptake (Kim et al., 2012) suggesting that P2Y2R up-regulation enhances microglia-dependent Aβ degradation. Interestingly, P2Y2R expression is up-regulated in 10 weeks old TgCRND8 AD mouse models and P2Y2R heterozygous mice showed accelerated pathology compared to wild-type mice (Ajit et al., 2014). In contradiction with data from the TgCRND8 mice but supporting the idea of P2Y2R displaying protective effect, data demonstrate a downregulation of P2Y2R in parietal cortex of AD patients (Lai et al., 2008). Discrepancies between mice and human P2Y2R expression regulation may be explained by the stage at which the studies were conducted. Altogether, these results suggest a protective role of P2Y2R signaling in the context of AD.

The other major P2 receptors are the ATP-gated ion channels P2X receptors. There are seven P2XR subunits whose assembly in trimers forms cation-permeable channels that are widely expressed in the CNS. To date, P2X4R and P2X7R are the only P2XRs pertinently characterized in microglia. P2X7 is the most studied in AD context because of its well-established pro-inflammatory role, being a key step in the NLRP3 inflammasome activation leading to IL-1β release (Bhattacharya and Biber, 2016; Adinolfi et al., 2018). P2X7R is up-regulated in AD patient brains (McLarnon et al., 2006; Martin et al., 2018) and administration of Aβ peptide in rat hippocampus increases P2X7R expression (McLarnon et al., 2006). Moreover, downregulating or blocking P2X7R decreases pro-inflammatory cytokines release in microglia cell cultures stimulated by ATP and Aβ (Ni et al., 2013). On the other hand, Martin et al. (2018) demonstrate that P2X7R deficiency ameliorates cognitive functions and reduces amyloid load without altering IL-1β level. This set of data suggest a pro-inflammatory role of P2X7R in AD. Lastly, the C489T polymorphism of P2X7R, which causes a loss of function, was found to be less frequent in AD patients, supporting a potential contribution of P2X7R in AD pathogenesis (Sanz et al., 2014).

Another highly expressed P2X receptor in reactive microglia is P2X4R. P2X4R are highly up-regulated upon microglia activation and have been implicated in neurological disorders and inflammatory processes (Suurväli et al., 2017). Although P2X4R is decreased in AD brain patients and neuronal P2X4R seems to modulate Aβ-induced neuronal death (Varma et al., 2009), no work has yet been performed on microglial P2X4R in AD.

The third main purinergic receptor family is that of adenosine P1Rs, which comprises A1, A2a, A2B, and A3 receptors. P1R are G protein-coupled receptors abundantly expressed in the CNS and all P1 subtypes are expressed in microglia. A1 and A2a receptor expressions have been found to be dysregulated in post-mortem tissues from AD patients (Erb et al., 2018), thus suggesting that P1 receptors might be implicated in AD. However, to date, not much is known regarding the contribution of microglial P1 signaling in the context of AD.

Overall, those few studies suggest the implication of microglia purinergic receptors in AD through different processes such as inflammation or phagocytosis. However, since purinergic receptors are expressed in all neural cell types incriminated in AD, more specific tools such as cell-specific KO models are required to decipher the roles of microglial purinergic signaling.

### Microglial Calcium Signaling, a Poorly Studied Messenger in AD

Calcium signaling is a key second messenger in almost all cell type and is essential for the normal CNS functioning. Many microglia functions are mediated by Ca^2+^ (McLarnon, 2005; Färber and Kettenmann, 2006). In particular, microglia reaction is accompanied with intracellular calcium increase, a process that is required to induce cytokines and chemokines release (Hoffmann et al., 2003).

Calcium signaling dysregulation in AD has been widely studied in neurons (Tong et al., 2018), but very little is known about calcium signaling in microglia in the context of AD. In an early study, Combs et al. (1999) demonstrated that stimulation of cultured microglia with Aβ_25__–__35_ peptide results in a transient increase in intracellular [Ca^2+^]. This calcium is released from intracellular store and leads to microglia activation and neurotoxic factors production. More recently, McLarnon et al. showed that cultured microglia from AD patients have higher basal Ca^2+^ level but lower amplitude and longer-lasting ATP-induced calcium response with respect to that measured in microglia from non-demented individuals, thus suggesting that calcium signaling is impaired in microglia from AD patients (McLarnon et al., 2005).

Altered P2YR-dependent calcium signaling has been observed in plaque-associated microglia in AD mouse models whereas plaque-distant microglia showed similar Ca^2+^ activity than matched controls (Brawek et al., 2014). As calcium mediates many microglial functions, it might be interesting to further investigate microglia calcium signaling in the context of AD. Particularly since some purinergic receptors are likely implicated in AD, it might be worth studying whether calcium signaling dysregulation involves purinergic signaling impairment.

## Microglia in Alzheimer Disease: Current Hot Topics

The involvement of microglia in AD is a relatively new area of research, but it is growing at a fast pace. In addition to the pathways described above, new areas of investigation are emerging or revisited based on our current knowledge of microglial functions. In the following part, we highlight few current hot topics (Figure 2). These new areas of research will help to increase our understanding of AD pathogenesis, and may, on a longer term, help to better stratify the patients and to design tailored therapeutic strategies.

### Sexual Dimorphism, a Key Factor to Design Efficient Therapeutic Strategies

The impact of sexual dimorphism in biology is a hot topic, and AD research makes no exception. Indeed, AD prevalence is two-fold higher in women (Hebert et al., 2013), and women suffering from AD display specific cognitive alterations, biomarker patterns or risk factors susceptibility (Ferretti et al., 2018). This sex effect was, at first, thought to be due to higher longevity but recent reports show that it is clearly not the only explanation (Viña and Lloret, 2010). The involvement of sex is still a subject of intense debate and biological mechanisms involved in human pathology are still controversial. Because most AD pre-clinical studies use either males or females, but rarely both, the cellular and molecular mechanisms involved in AD sexual dimorphism are still poorly understood. Yet, the few studies comparing the influence of sexual dimorphism in AD mouse models, all agree on a sooner precocious and stronger AD phenotype in females (Table 1). Sex-based effects are observed in different AD mouse models, both at behavioral and histological levels. Globally, cognitive alterations in females appear at younger ages and remain stronger than in males (Clinton et al., 2007; Carroll et al., 2010; Gallagher et al., 2013; Yang et al., 2018). Whatever brain areas, models and ages, females display more amyloid plaques and higher amount of soluble Aβ (Wang et al., 2003; Carroll et al., 2010; Gallagher et al., 2013; Ben Haim et al., 2015; Janus et al., 2015; Jiao et al., 2016; Yang et al., 2018). Furthermore, in late stages, Tau phosphorylation levels and number of Phospho-Tau positive cells are higher in females (Clinton et al., 2007). Synaptic and neuronal degeneration processes seem also to be stronger in aged females (Jiao et al., 2016). Relative to neuroinflammation, glial cells from old female AD mice present stronger reactive states compared to males and are associated with higher levels of pro-inflammatory factors (Jiao et al., 2016; Yang et al., 2018). While sex effects are clearly established in both AD mice and patients, it remains unknown whether these modifications are due to systemic/hormonal effects or whether it also exists at the cellular level.

**TABLE 1:** Impact of sexual dimorphism on AD pathophyiology in various mice models.

**AD mice model**	**Age**	**Brain area**	**Studied parameter (readout)**	**Sex influence**	**References**
**Behavior studies**					
3xTg	6–9 mo	–	MWM learning deficit	♀ only	Clinton et al., 2007
	15mo	–	MWM learning deficit	♀ = ♂	Clinton et al., 2007
	2–6–9–15mo	–	NOR	♀ = ♂	Clinton et al., 2007
	12mo	–	MWM retention task	♀ < ♂	Yang et al., 2018
	12–14mo	–	Spontaneous alternation	♀ < ♂	Carroll et al., 2011
**Amyloid beta cascade**					
3xTg	2–6–12–15mo	Brain homogenate	Aβ 40/42 soluble and insoluble fractions (ELISA)	♀ = ♂	Clinton et al., 2007
	6–8mo	PFCx	Plaques density (Aβ antibody)	♀ > ♂	Carroll et al., 2011
	12mo	SB, CA1, PFCx	Plaques density (Aβ antibody)	♀ > ♂	Carroll et al., 2011
	12mo	HC	Aβ positive area (6E10 staining)	♀ > ♂	Yang et al., 2018
APP(swe)/PS1(de9)	4–12–17mo	HC	Aβ 40/42 (ELISA)	♀ > ♂	Wang et al., 2003
	12–17mo	HC	Plaque area and density (W0–2 staining)	♀ > ♂	Wang et al., 2003
	?	HC, Cx	Plaque density (Congo Red staining)	♀ > ♂	Gallagher et al., 2013
	?	Brain homogenate	Insoluble Aβ42 (ELISA)	♀ > ♂	Gallagher et al., 2013
	?	Brain homogenate	Soluble Aβ42 (ELISA)	♀ = ♂	Gallagher et al., 2013
	?	Brain homogenate	*Ide* (mRNA expression)	♂ > ♀	Gallagher et al., 2013
	?	Brain homogenate	*Bace-1* (mRNA expression)	♀ > ♂	Gallagher et al., 2013
	12mo	OB, HC, NC	Plaque area and density (Congo red compared to Thioflavin-S and 6E10 staining)	♀ > ♂	Jiao et al., 2016
	12mo	Brain homogenate	Aβ 40/42 (Western Blot and ELISA)	♀ > ♂	Jiao et al., 2016
	18mo	HC, Cx	Plaque area (Campbell-Switzer Silver staining)	♀ > ♂	Janus et al., 2015
**Tau load**					
3xTg	6mo	HC	Tau positive region (HT7 antibody)	♀ = ♂	Clinton et al., 2007
	12mo	HC	Number of P-Tau positive neurons and P-Tau area (pT231-Tau antibody)	♀ > ♂	Yang et al., 2018
APPswe/PS1de9	12mo	CA1, CA3, NC	P-Tau positive area (IHC) and P-Tau quantity (WB)	♀ > ♂	Jiao et al., 2016
**Inflammation**					
3xTg	12mo	HC	IBA1 and GFAP positive area	♀ > ♂	Yang et al., 2018
APPswe/PS1de9	12mo	Brain homogenate	TNF, IFN and Il-1β protein levels (ELISA)	♀ > ♂	Jiao et al., 2016
	12mo	OB, HC, NC	IBA1 and GFAP positive area	♀ > ♂	Jiao et al., 2016
	12mo	HC, NC	Caspase-3 positive neurons	♀ > ♂	Jiao et al., 2016
**Neurodegeneration**					
APPswe/PS1de9	12mo	CA1, CA3, NC	Number of neurons	♂ > ♀	Jiao et al., 2016
	12mo	Brain homogenate	Synaptic proteins expression level	♂ > ♀	Jiao et al., 2016
	10mo	Whole Brain	White matter, axonal and myelinated fibers volumes	♀ > ♂	Zhou et al., 2018
**Comorbidity**					
APPswe/PS1de9	12mo	Whole Brain	Microhemorrage number	♀ > ♂	Jiao et al., 2016

*This table is a non exhaustive compilation of the literature concerning AD sexual dimorphism. NC for Neocortex, PFCx for PreFrontalCortex, HC for Hippocampus, SB for Subiculum, OB for Olfactory Bulb, MWM for Morris Water Maze, IDE for Insulin Degrading Enzyme, ? for missing data.*

A growing number of studies highlights the impact of sexual dimorphism on microglia shape and functions (Table 2). Many sex mediated effects on microglia have been demonstrated. They seem to be highly dependent on age and brain region: (1) in both mice and rats, adult males show higher microglia densities (Guneykaya et al., 2018; Perkins et al., 2018); (2) adult females microglia also display higher proportion of cells with long and thick processes while male microglia have larger soma (Schwarz et al., 2012; Guneykaya et al., 2018; Weinhard et al., 2018). These differences may indicate that adult female microglia are in a more homeostatic state while males display a more reactive state. Functional differences are also observed, with microglia from male brain displaying higher migration rates while female’s present stronger phagocytic activity related to higher expression of phagocytosis associated genes (Nelson et al., 2017; Yanguas-Casás et al., 2018). RNA sequencing also revealed a more protective transcriptomic profile for female microglia while male microglia displayed a more inflammatory phenotype (Villa et al., 2018). A recent study also reports a sex impact on microglial electrical properties, suggesting that sex may affect microglia secretory profile, a property that can directly influence inflammatory response abilities (Guneykaya et al., 2018). It was initially thought that microglia sex differences depend from circulating sexual hormones (Nissen, 2017), but this view was recently challenged as it was shown that female microglia retain their protective properties when transplanted in males brain (Villa et al., 2018).

**TABLE 2:** Impact of sexual dimorphism on microglial shape and function.

**Parameter**	**Read out**	**Brain area/cellular model**	**Age**	**Sex influence**	**References**
**Microglia density**					
Microglia density	IBA1 positive cells	Hippocampus	P21	♂ > ♀	Guneykaya et al., 2018
		Amygdala	P21	♀ > ♂	
		Cx, HC, and Amygdala	13w	♂ > ♀	
		HC, Amygdala, and Striatum	3mo	♂ > ♀	Perkins et al., 2018
**Shape**					
Microglial compartment volume	IBA1 and CD68 staining	CA1	P8	♀ > ♂	Weinhard et al., 2018
			P28	♂ > ♀	
	Soma size	Cx, HC, and Amygdala	13w	♂ > ♀	Guneykaya et al., 2018
Microglia ramification complexicity	IBA1 positive cells: Ameboid	CA3, Dentate Gyrus, and Amygdala	P0	♀ > ♂	Schwarz et al., 2012
		CA1, CA3, Dentate Gyrus, Parietal Cortex, and Amygdala	P4	♂ > ♀	
	IBA1 positive cells: Ramified (thick and long processes)	CA3, Dentate Gyrus, Parietal Cortex, and Amygdala	P30 and P60	♀ > ♂	
**Function**					
Cell migration	Transwell migration	Primary microglia culture	P0 to P2	♂ > ♀	Yanguas-Casás et al., 2018
Phagocytosis	Phagocytic cup (CD68+, Dapi+)	HC	Neo-natal	♀ > ♂	Nelson et al., 2017
	Latex beads	Acute cortical and hippocampal slices	13w	♀ = ♂	Guneykaya et al., 2018
	Fluorescent beads	Primary microglia culture	P0 to P2	♀ > ♂	Yanguas-Casás et al., 2018
Electrophysiological properties	Baseline inward and outward conductance	Microglia located in layers 2–6 of the somatosensory cortex in acute slices	N/A	♂ > ♀	Guneykaya et al., 2018
	Resting membrane potential		N/A	♂ > ♀	
Inflammatory profile (protein level)	Il-10	Cx and HC	P60	♀ = ♂	Schwarz et al., 2012
	Il-1b	Cx and HC	P60	♀ > ♂	
Inflammatory profile. (gene expression)	Il-10, Il1r1, Il1f1 genes	Cx and HC	P0, P4, P60	♀ > ♂	Schwarz et al., 2012
	RNASeq	Isolated microglia	3mo	♀: protective	Villa et al., 2018
				♂: Inflammatory	

*This table is a non exhaustive compilation of the literature concerning microglial sexual dimorphism. Cx for Cortex, HC for Hippocampus, N/A for missing data.*

Thus, although the molecular pathways involved in this microglial sexualization remain largely unknown, the current data suggest that, at least in adulthood, female microglia are in a more homeostatic and protective state. In the context of AD, it could be speculated that female microglia committed to homeostatic functions would need longer time exposure or stronger stimuli to polarize their shape and functions to answer correctly to harmful stimuli. Additionally, when they finally react to Aβ, they will change their local environment from a low- to a high-inflammatory environment. This drastic change could lead to more harmful effects on microglia themselves but also on all other surrounding cells and may explain why female are more likely to develop the pathology.

Although the effect of sex on microglial functions has been implicated in various CNS pathologies (Sorge et al., 2015; Charriaut-Marlangue et al., 2018; Jullienne et al., 2018; Mapplebeck et al., 2018; Thion et al., 2018), further studies are needed to understand the impact of microglial sexual dimorphism in AD initiation and progression and to which extend it relies to sex disbalance in AD. Whether sexual dimorphism mechanisms are similar in human and animal models also need to be clarified.

### Microglial Diversity Is Both a Challenge and an Opportunity

Microglia cell diversity has been recognized since their first description by Pio del Rio-Hortega in 1919 (Sierra et al., 2016), who reported the existence of different morphological microglia phenotypes in the human brain. He also established that microglia morphology is considerably altered in disease conditions, introducing the concept that microglia tightly adapt to their local environment. More recent functional, morphological, immunohistochemical, and medium throughput analyses of microglia in pathological conditions also pointed out to the existence of a diversity of reactive phenotypes (Hanisch and Ketternmann, 2007; Ransohoff and Perry, 2009; Kierdorf and Prinz, 2017). In this respect, several semi-automated tools are now available to enable the identification of phenotypically distinct microglial cell subpopulations (Wagner et al., 2013; Verdonk et al., 2016; Salamanca et al., 2019).

In the last decade, the development of high throughput transcriptomic approaches combined with improved cell purification techniques helped refine our understanding of the molecular diversity of microglia both in physiological (Grabert et al., 2016) and pathophysiological conditions (Hirbec et al., 2017; Holtman et al., 2017; Sousa et al., 2017; Dubbelaar et al., 2018). They established that microglia cannot be categorized in a discrete number of physiological and pathological states. However, the breakthrough in the appreciation of the microglia diversity arose from the emergence of single-cell high throughput approaches. In particular single-cell RNAseq (scRNA-seq) enables investigating the diversity at cellular resolution and allows a detailed examination of cell states diversity and changes that are reflective of those *in vivo* (Macosko et al., 2015).

Two very recent scRNA-seq studies established the spatial and temporal diversity of microglia during the mouse development, and in either neurodegenerative or inflammatory conditions (Hammond et al., 2019; Masuda et al., 2019). These studies, together with more focused previous ones, (Keren-Shaul et al., 2017; Friedman et al., 2018) established that different microglia subpopulations co-exist at a given physiological or pathological state. Such heterogeneity within the microglia cell population represents both a challenge and an opportunity: the existence of distinct subpopulations supports the design of specific treatments targeting specific subpopulations with the aims of either promoting the beneficial subpopulations and/or hampering the deleterious ones.

What about microglia diversity in AD? It is known for years that AD brains exhibit at least two very distinct morphological microglia phenotypes: cells associated to the amyloid plaques display a “reactive”/amoeboid-like phenotype whereas those present in the rest of the parenchyma have a homeostatic-like morphology (Krabbe et al., 2013). As a mark of the importance of this diversity, it was shown that transcripts up-regulated in microglia isolated from AD mice were more highly expressed in tissues isolated in the vicinity of amyloid plaques compared to those distant from plaques (Orre et al., 2014). This suggested that microglia associated to plaques activate specific signaling pathways and functions. Using scRNA-seq of sorted 5xFAD mouse brain immune cells, Keren-Shaul et al. (2017) identified three distinct subtypes of microglia, including DAMs whose relative abundance increases with disease progression and which are preferentially located around amyloid plaques. The translational relevance of the DAMs was confirmed in human postmortem brains in which specific DAM markers are expressed in a subset of microglia in AD patients, but not in control subjects. More recently, in the knock-in APP^NLFG^ AD mouse model (Saito et al., 2014; Sala Frigerio et al., 2019) identified activated- and interferon response microglia (ARM and IRM, respectively). ARMs partly overlap with DAMs, but by applying cell trajectory inference methods, these last authors demonstrated that ARMs were not solely present in disease conditions and are part of the normal brain aging process. However, many unresolved questions remain regarding the functional significance of this diversity. In particular, when it arises during AD progression and whether it is already present at the prodromal stages of the disease. With progress of both technology and analytic methods, it is now feasible to get a deeper understanding of the microglia diversity across AD progression. Identification of specific, potentially small in size, microglia subpopulations may have important implications for translational applications. Identification of factors released by these subpopulations and that can diffuse to the CSF may lead to the identification of new biomarkers. Additionally, identification of specific subpopulations will help discover new pathways and functions that contribute to the disease progression and may lead to the development of more specific functional positron emission tomography (PET) tracers. Deciphering, whether these subpopulations have beneficial, neutral or detrimental effects on brain cells, including neurons, will open the way for the design innovative disease-modifying therapeutic strategies.

### Studying AD at Early Stages to Identify Early Biomarkers

After years of unsuccessful clinical trials, scientists are now realizing that AD is a pathology we may want to prevent (or stop) rather than to cure. This is calling for the identification of early diagnostic biomarkers, which are sorely lacking to help prodromal diagnostic, or design innovative disease-modifying therapeutic strategies. Although AD early stages are still less studied, increasing number of studies are now focusing on the prodromal phases to unravel early dysregulations.

Because one of microglia’s main role is to sense their environment and to react to danger, they are likely to play key roles in initial brain response to AD-driven changes. In agreement, a recent PET and magnetic resonance imaging (MRI) based study showed that AD patients have a first microglial activation peak before they display any other hallmarks of the pathology, suggesting that microglial dysfunction is critically involved in AD initiation (Fan et al., 2017). The involvement of microglia in AD initiation stages raises the question of which microglial targets could be modulated at an early stage to slow down the progression of the disease.

*Extracellular vesicles* (EVs) are a family of small membrane vesicles, which includes exosomes and microvesicles (MVs), transporting various types of molecules. In the recent years, EVs have emerged as a new mean of communication between cells. Microglia abilities to secrete EVs and MVs are part of their essential inflammatory functions. In the Aβ phagocytosis process, overloaded microglia can release Aβ-containing small secretory vesicles (Joshi et al., 2014), as well as Tau or P-Tau through exosomes (Saman et al., 2012; Asai et al., 2015). Accordingly, the levels of myeloid MVs detected in the CSF of MCI or early stage AD patients, is correlated with the extent of their white matter damage (Agosta et al., 2014). Microglial EVs may thus be regarded as a possible mean to spread the pathology during early stages. Interestingly, because they are secreted and can access first the CSF compartment and then the blood, microglial EVs may represent valuable diagnosis tools (Trotta et al., 2018).

*Oxidative stress* is known as a necessary but potentially harmful process. In response to fibrillary Aβ, reactive microglia produce free radicals, notably superoxide via the NADPH oxidase (NOX) (Harrigan et al., 2008). Moreover, NOX activity is increased in MCI patients compared to controls (Bruce-Keller et al., 2010). These findings open the way for using NOX activity as the marker of early microglia reaction in AD. Interfering with NOX activity through specific compounds may also provide a mean to slow down the pathology (Dumont and Beal, 2011).

*Iron* is an important metal implicated in vital biological processes. However, intracellular iron overload can lead to neuronal degeneration (Zhang et al., 2014). Microglia are able to interact, transport and metabolize iron. When they secrete pro-inflammatory factors, microglia can stimulate neuronal iron uptake leading to an increase in neurodegeneration (Zhang et al., 2014). Interestingly, patients with specific iron overload disorder are subject to earlier onset of AD, suggesting that iron may be involved in the initiation steps of the pathology. Moreover, iron chelation seems to be beneficial to AD patients. Further work is needed to understand the early implication of microglial iron regulations in AD (Nnah and Wessling-Resnick, 2018).

## Barriers to Discoveries

Although fundamental knowledge is of importance, the ultimate goal of characterizing cells functions and dysfunctions in physiological and pathological conditions is to design efficient therapeutic strategies with clinical benefit. Understanding the complexity of Alzheimer’s disease is one of the XXIth century challenges. To do so we mainly rely on animal models that mimic the symptoms of the pathology (amyloid deposits, hyperphosphorylated tau, cognitive alterations, etc.). However, so far, the results generated from these models have generally failed to translate to clinic, and only symptomatic and poorly efficient treatments exist (Ransohoff, 2018). In the two following paragraphs, we explore two important factors that may have been under-estimated in AD pre-clinical research: the relevance of current AD mouse models and the immunological differences between mice and humans. These remarks do not only apply to the study of microglia’ roles in AD, but are important issues to consider for the design of any preclinical studies.

### Are the Current Mouse Models Tailored to Understand AD and Identify Drug Targets?

Most of the animal models are genetically engineered to overexpress human protein mutations also leading to an overexpression of their respective by-products, thus generating potential confounding factors (Sasaguri et al., 2017). In addition, most AD models are based on familial mutations whereas early-onset familial AD is estimated to account for only 3.5% of total AD cases (Harvey et al., 2003). In addition, some of these models are based on a combination of mutations that were never found in patients. Because it is the first hallmark of AD, most of the models focused on the amyloidogenic pathway (Sasaguri et al., 2017). However, it is clear that, in humans, there is a concomitant effect of Aβ and P-Tau. To solve this issue, the 3x-Tg model is combining Aβ and Tau associated features. However, it only poorly represents the pathology in term of kinetic of appearance of the symptoms as cognitive deficits appear way too early and before any amyloid accumulation or Tau dysregulation (AlzForum^1^). To solve these issues, new mouse-based or human-based models are created (see section “New opportunities in AD research”).

To study microglia impact on AD, new models have been created by combining microglial dedicated tools with AD models (Zhou et al., 2018). One of the most commonly used models is the CX3CR1^+/eGFP^ x APP/PS1 strain, in which an allele of the fractalkine receptor CX3CR1 is replaced by a GFP allowing to track microglial cells. However, to our knowledge, the impact of the CX3CR1 haplo-deficiency on AD development has not yet been thoroughly investigated. Finally, AD studies are generally conducted in old mice, when the pathology symptoms and hallmarks are well established. This stage of the pathology is probably too advanced for the identification of efficient disease modifying drugs.

### Human and Mice Are Not Immunologically Similar

In the field of inflammation, the comprehensive study by Seok et al. (2013) revealed that gene dysregulation in mouse models of severe human inflammatory conditions (endotoxemia, burns, and trauma) do not correlate with human genomic changes. This raises the question of the relevance of mouse models to study the role of immune cells in diseases, including brain disease. Several factors known to be of importance for immune functions differ between rodents and human studies. Most often, rodent models and studies use inbred strains whereas human genetic backgrounds are much more diverse. Additionally, humans are exposed to multiple diseases whereas research mouse models are raised in tightly controlled environment (Davis, 2012).

Functional studies investigated the response of human microglia to either endogenous (i.e., M-CSF) or exogenous (i.e., LPS) stimuli (Melief et al., 2012; Smith et al., 2013). For the most part, they show comparable results with mouse analyses, thus agreeing with two comprehensive transcriptomic studies which compared gene expression profiles in human and mouse microglia and concluded that, overall, gene expression is very similar in the two species (Galatro et al., 2017; Gosselin et al., 2017). Of interest to brain diseases, a good correlation between human and mouse microglia in response to neurodegeneration was also observed by other authors (Holtman et al., 2015; Keren-Shaul et al., 2017; Krasemann et al., 2017).

However, notwithstanding their global resemblance, significant number of genes are differentially expressed in mouse and human microglia. In particular, specific immune genes are only expressed in human samples (Gosselin et al., 2017). In addition, differences in the relative expression of lineage- and signal-dependent transcription factors between mice and humans were observed (Holtman et al., 2017). In line, previous studies identified several molecular pathways and signaling functions that significantly differ between mouse and human microglia. This includes proliferation, response to TGFβ1, Siglecs signaling, nitric oxide (NO) production, and response to certain drugs such as valproic acid (VPA) (Smith and Dragunow, 2014).

Of relevance to AD, Siglec-3 (CD33) that has been identified as a risk factor for AD (Bertram et al., 2008) displays substantial species differences in expression patterns and ligand recognition (Lajaunias et al., 2005). Additionally, age-related changes of immune and cognitive functions may not be correctly modeled in rodents, whose life expectancy is far less compared to humans. Accordingly, gene expression changes in aged human brain are significantly different from those in the mouse brain (Loerch et al., 2008; Bishop et al., 2010). In agreement, Galatro et al. (2017) revealed that there is a limited overlap in age-related changes in human and mouse microglia, highlighting that data obtained in aged mice should be extrapolated to the human situation with caution. Translation of results from mice to humans is also hampered by the lack of tools to precisely characterize microglial reactivity in patients: TSPO binding is so far the only way to study microglia reaction in a clinical context.

Although methods to purify, culture and experiment on human microglia have been established, access to human samples that meet the requirements of high-quality studies is limited. AD mouse models are thus undoubtedly useful to decipher the roles of specific microglial functions and signaling pathways. However, the species differences highlighted above should prompt scientists to confirm the results they obtained in mouse models on human samples.

## New Opportunities in AD Research

While current models to study AD have multiple limitations, scientists are trying to generate new models with limited pitfalls. In this last part, we are reviewing new approaches currently developed. These approaches are expected to improve our knowledge on microglial functions and help bridge the gap between *in vitro* studies, rodent models and the human disease.

### Promising Animal Models to Overcome Identified Pitfalls

Although mouse models present significant pitfalls, they are attractive models in preclinical studies. Indeed, they are small, easy to raise, mammals with reasonable life expectancy. They reproduce well in captivity and can be engineered and/or humanized in a relatively easy way. Furthermore, with the exponential development of a wide repertoire of mouse lines (Model Organism Development and Evaluation for Late-Onset Alzheimer’s Disease^2^), it is possible for scientists to explore new hypotheses by combining different mouse lines. In this context and to overcome the problems associated with APP overexpression, Drs Saito and Saido developed new knock-in AD mouse models, namely the APP^NLF^ and APP^NLFG^ mice (Saito et al., 2014). In these models, mutated APP is expressed from its endogenous promoter, leading to a kinetic of appearance of the symptoms which is more comparable with human pathology (Sakakibara et al., 2018). These models are not thoroughly characterized yet and still excludes Tau-associated dysfunctions. However, in the APP^NLF^ model, the delay before Aβ deposits appearance supports that it may represent an interesting model to study the prodromal phase of the disease.

Other models, not based on genetic modifications, have also emerged in the last decades. Compared to humans, dogs share an almost identical enzymatic machinery to process APP. Moreover, they may naturally develop an age-related cognitive dysfunction that reproduces several aspects of AD (Schmidt et al., 2015). With its high life span, the canine model can be useful specifically to study pathways involved in Aβ deposition and clearance in the early phases of the disease (Sarasa and Pesini, 2009). To get closer to human pathology, several primate models are currently used to study AD. Depending on their phylogenetic distance to humans, they neither display the same kinetic nor kind of neurological alterations (Heuer et al., 2012). Among primates, *Microcebus Murinus* also known as the gray mouse lemur, is particularly interesting (Bons et al., 2006). Indeed, they are small animals, just over the size of a mouse. They can be raised in cohorts of several individuals, and can reproduce in captivity. Along aging some individuals naturally develop an AD like pathology with Aβ plaques (Mestre-Francés et al., 2000), associated with specific transcriptomic remodeling (Abdel Rassoul et al., 2010). Histological features also seem to be associated with cognitive alterations (Trouche et al., 2010). Although we know that, at least in the spinal cord, the gray mouse lemur microglial shape and distribution is closer to human compared to mouse (Le Corre et al., 2018), microglia are still poorly studied in this model. Further studies are needed to investigate the involvement of microglia in this age-related AD like pathology.

Although, both dog and primate models represent interesting models, they are not so easy to use. For example, there are only few breeding centers for *Microcebus Murinus* worldwide. Those animals also need more housing space and their use is subjected to drastic ethic rules. Moreover, many of the research tools are designed on mouse genome/proteins limiting the use of these peculiar models.

### hiPSCs-Derived Microglia: An Emerging Tool to Decipher the Role of Microglia in AD

To overcome species issues, microglial cell lines of human origin can be useful. They allow to study specific biological functions or functional pathways; however, they cannot reproduce the complexities of cells. This particularly true for cells which, like microglia, are highly adapted to their environment. Bone marrow-derived or blood monocytes-derived macrophages represent another source of human microglia-like cells. However, because these cells are of fundamentally different embryonic origin (Hoeffel and Ginhoux, 2015), they are likely poor models of adult microglia. Adult primary microglia can be harvested from dissociated brain area, but this process generally lead to low yields and can only sparsely be obtained for human samples. Moreover, isolation processes and culturing methods impact on gene expression pattern thus calling for caution in interpreting results based on these approaches (Gosselin et al., 2017).

An alternative to those issues is the development of cell reprograming methods, which offer the possibility to generate human pluripotent cell lines (hiPSCs) from healthy individuals but also from patients with specific diseases (Sullivan and Young-Pearse, 2017). Human iPSCs-derived neural cells can be produced in consistent yields. In the context of AD, iPSC-derived neurons issued from either familial AD (FAD) patients carrying mutations found in AD or sporadic AD (SAD) patients recapitulate some of the main hallmarks of the disease (Poon et al., 2017). Similarly, astrocytes derived from AD-patient iPSC show significant morphological and functional alterations, including up-regulation of inflammatory cytokines (Chandrasekaran et al., 2016; Auboyer et al., 2019).

Human pluripotent cell lines have recently been successfully differentiated into microglia (Muffat et al., 2016; Abud et al., 2017; Douvaras et al., 2017; Haenseler et al., 2017; Takata et al., 2017; Pocock and Piers, 2018). Although there is yet no consensus protocol to obtain hiPSCs-derived microglia, all protocols follow similar steps based on this cell type ontogeny. Based on such protocol, Garcia-Reitboeck et al. (2018) recently described microglial dysfunctions in hiPSCs-derived microglia from TREM2 T66M and W50C carriers, thus outlining the interest of such iPSCs approaches to decipher the role of microglia functions and dysfunctions in AD. Deciphering to which extend hiPSCs microglia derived from different patients (i.e., different SADs and/or different FAD mutations) share the same molecular and/or functional characteristics will help better understand the heterogeneity of AD, and may open the way toward the use of hiPSCs to implement personalized treatment in AD.

An important emerging concept is that, because microglia are CNS resident macrophages highly adapted to the CNS environment, a two-steps protocol is required to obtain hiPSCs-derived microglia with genuine microglial functions. In this scheme, the first step is to obtain iPSCs-derived macrophages progenitors and second to grow them in a conditioned environment, i.e., in the presence of other neural cells (Lee C.Z.W. et al., 2018). Under such experimental settings, hiPSCs-derived microglia exhibit phenotype, gene expression profile and functional properties close to brain-isolated microglia (Pandya et al., 2017; Takata et al., 2017).

Maintenance of tissue architecture is an important aspect of *in vivo* studies. Interestingly, hiPSCs derived cells can be grown in 3D cultures to model the cytoarchitecture and the connectivity of the brain while allowing *in vitro* manipulation and experimentation. Such 3D or organoids models open the way for studying human microglia under conditions that are close to physiological and physio-pathological context. Although specific pitfalls exist with such approaches, including the need to develop isogenic controls to alleviate the risk of cofounding factors due to difference in genetic backgrounds between patients and controls, there is little doubt that these approaches will be instrumental to decipher the role of microglia in the progression of AD.

## Concluding Remarks

Glial cells, including microglia, have long been suspected to play a role in Alzheimer’s disease but only because of their ability to react to neuronal dysfunctions (e.g., Amyloid and Tau aggregates). This neurocentric view, which considered glial cells as secondary, has been challenged recently by the results of genetic association studies identifying genetic loci associated with risk of Alzheimer’s that are associated to genes preferentially or exclusively expressed in glial cells (Verheijen and Sleegers, 2018). This has refined our view of how Alzheimer disease initiates and progresses, and introduced new concepts and ideas for Alzheimer’s pathophysiological mechanisms, both at the molecular and cellular levels.

Because of their abilities to sense and react to their environment, reactive microglia are likely playing key early roles in the disease progression and may lead to the identification of early biomarkers. Because they can drive functional changes in astrocytes (Liddelow and Barres, 2017) and crosstalk with non-neuronal immune cells (Dionisio-Santos et al., 2019), they also represent attractive drug targets to stop or limit the disease progression.

As reported here, the exact contribution of the different reactive microglia subtypes to AD is currently unclear and the subject of intense researches. Over the recent years, several technological breakthroughs have been achieved, allowing scientists to address new challenging questions. These technical developments now allow studying microglia roles with medium or high throughput flows, and perform fine analysis of their functions in preserved environments. A better understanding of the contribution of microglia cells to AD initiation and progression is expected to renew the interest of big pharma to re-invest in the field and will pave the way toward better designed strategies.

Many factors need to be considered, including sex, age, species, molecular diversity, health status, communication with the periphery, etc., to fully decipher the role of microglia in AD. These are undoubtedly challenging but also very exciting fields of research, which hold the promise of defining innovative therapeutic strategies and reduce the socio-economic burden of this devastating disease.

## Author Contributions

A-LH, JH, LU, and HH designed and drafted the sections of the manuscript. A-LH prepared the tables and the figures.

## Conflict of Interest Statement

The authors declare that the research was conducted in the absence of any commercial or financial relationships that could be construed as a potential conflict of interest.

## Footnotes

1 https://www.alzforum.org/

2 https://model-ad.org

## References

[B1] Abbracchio M. P. Ceruti S. (2007). P1 receptors and cytokine secretion. *Purinergic Signal.* 3 13–25. 10.1007/s11302-006-9033-z 18404415 PMC2096764

[B2] Abdel Rassoul R. Alves S. Pantesco V. De Vos J. Michel B. Perret M. (2010). Distinct transcriptome expression of the temporal cortex of the primate *Microcebus murinus* during brain aging versus Alzheimer’s disease-like pathology. *PLoS One* 5:e12770. 10.1371/journal.pone.0012770 20862281 PMC2940844

[B3] Abud E. M. Ramirez R. N. Martinez E. S. Healy L. M. Nguyen C. H. H. Newman S. A. (2017). iPSC-derived human microglia-like cells to study neurological diseases. *Neuron* 94 278–293.e9. 10.1016/j.neuron.2017.03.042 28426964 PMC5482419

[B4] Adinolfi E. Giuliani A. L. De Marchi E. Pegoraro A. Orioli E. Di Virgilio F. (2018). The P2X7 receptor: a main player in inflammation. *Biochem. Pharmacol.* 151 234–244. 10.1016/j.bcp.2017.12.021 29288626

[B5] Agosta F. Dalla Libera D. Spinelli E. G. Finardi A. Canu E. Bergami A. (2014). Myeloid microvesicles in cerebrospinal fluid are associated with myelin damage and neuronal loss in mild cognitive impairment and Alzheimer disease. *Ann. Neurol.* 76 813–825. 10.1002/ana.24235 25087695

[B6] Ajami B. Bennett J. L. Krieger C. Tetzlaff W. Rossi F. M. (2007). Local self-renewal can sustain CNS microglia maintenance and function throughout adult life. *Nat. Neurosci.* 10 1538–1543. 10.1038/nn2014 18026097

[B7] Ajit D. Woods L. T. Camden J. M. Thebeau C. N. El-Sayed F. G. Greeson G. W. (2014). Loss of P2Y2 nucleotide receptors enhances early pathology in the TgCRND8 mouse model of Alzheimer’s disease. *Mol. Neurobiol.* 49 1031–1042. 10.1007/s12035-013-8577-5 24193664 PMC3954904

[B8] Alzheimer A. (1907). über eine eigenartige Erkrankung der Hirnrinde. *Allg. Zeitschrift für Psychiatr. und phychish-Gerichtliche Medizin* 64 146–148.

[B9] Asai H. Ikezu S. Tsunoda S. Medalla M. Luebke J. Haydar T. (2015). Depletion of microglia and inhibition of exosome synthesis halt tau propagation. *Nat. Neurosci.* 18 1584–1593. 10.1038/nn.4132 26436904 PMC4694577

[B10] Auboyer L. Monzo C. Wallon D. Rovelet-Lecrux A. Gabelle A. Gazagne I. (2019). Generation of induced pluripotent stem cells (IRMBi001-A) from an Alzheimer’s disease patient carrying a G217D mutation in the PSEN1 gene. *Stem Cell Res.* 34:1013183. 10.1016/j.scr.2018.101381 30677723

[B11] Basilico B. Pagani F. Grimaldi A. Cortese B. Di Angelantonio S. Weinhard L. (2019). Microglia shape presynaptic properties at developing glutamatergic synapses. *Glia* 67 53–67. 10.1002/glia.23508 30417584

[B12] Bedner P. Dupper A. Huttmann K. Muller J. Herde M. K. Dublin P. (2015). Astrocyte uncoupling as a cause of human temporal lobe epilepsy. *Brain* 138 1208–1222. 10.1093/brain/awv067 25765328 PMC5963418

[B13] Belinson H. Michaelson D. M. (2009). ApoE4-dependent Aβ-mediated neurodegeneration is associated with inflammatory activation in the hippocampus but not the septum. *J. Neural Transm.* 116 1427–1434. 10.1007/s00702-009-0218-9 19370389

[B14] Ben Haim L. Carrillo-de Sauvage M.-A. Ceyzériat K. Escartin C. (2015). Elusive roles for reactive astrocytes in neurodegenerative diseases. *Front. Cell. Neurosci.* 9:278. 10.3389/fncel.2015.00278 26283915 PMC4522610

[B15] Bertram L. Lange C. Mullin K. Parkinson M. Hsiao M. Hogan M. F. (2008). Genome-wide association analysis reveals putative Alzheimer’s disease susceptibility loci in addition to APOE. *Am. J. Hum. Genet.* 83 623–632. 10.1016/j.ajhg.2008.10.008 18976728 PMC2668052

[B16] Bhattacharya A. Biber K. (2016). The microglial ATP-gated ion channel P2X7 as a CNS drug target. *Glia* 64 1772–1787. 10.1002/glia.23001 27219534

[B17] Bishop N. A. Lu T. Yankner B. A. (2010). Neural mechanisms of ageing and cognitive decline. *Nature* 464 529–535. 10.1038/nature08983 20336135 PMC2927852

[B18] Bons N. Rieger F. Prudhomme D. Fisher A. Krause K.-H. (2006). *Microcebus murinus*: a useful primate model for human cerebral aging and Alzheimer’s disease? *Genes Brain Behav.* 5 120–130. 10.1111/j.1601-183X.2005.00149.x 16507003

[B19] Bouchon A. Hernández-Munain C. Cella M. Colonna M. (2002). A Dap12-mediated pathway regulates expression of Cc chemokine receptor 7 and maturation of human dendritic cells. *J. Exp. Med.* 194 1111–1122. 10.1084/jem.194.8.1111 11602640 PMC2193511

[B20] Brawek B. Schwendele B. Riester K. Kohsaka S. Lerdkrai C. Liang Y. (2014). Impairment of in vivo calcium signaling in amyloid plaque-associated microglia. *Acta Neuropathol.* 127 495–505. 10.1007/s00401-013-1242-2 24407428

[B21] Bruce-Keller A. J. Gupta S. Parrino T. E. Knight A. G. Ebenezer P. J. Weidner A. M. (2010). NOX activity is increased in mild cognitive impairment. *Antioxid. Redox Signal.* 12 1371–1382. 10.1089/ars.2009.2823 19929442 PMC2864654

[B22] Burguillos M. A. Deierborg T. Kavanagh E. Persson A. Hajji N. Garcia-Quintanilla A. (2011). Caspase signalling controls microglia activation and neurotoxicity. *Nature* 472 319–324. 10.1038/nature09788 21389984

[B23] Burnstock G. (2016). An introduction to the roles of purinergic signalling in neurodegeneration, neuroprotection and neuroregeneration. *Neuropharmacology* 104 4–17. 10.1016/j.neuropharm.2015.05.031 26056033

[B24] Butovsky O. Jedrychowski M. P. Moore C. S. Cialic R. Lanser A. J. Gabriely G. (2014). Identification of a unique TGF-β–dependent molecular and functional signature in microglia. *Nat. Neurosci.* 17 131–143. 10.1038/nn.3599 24316888 PMC4066672

[B25] Calovi S. Mut-Arbona P. Sperlágh B. (2019). Microglia and the Purinergic Signaling System. *Neuroscience* 405 137–147. 10.1016/j.neuroscience.2018.12.021 30582977

[B26] Cardona A. E. Pioro E. P. Sasse M. E. Kostenko V. Cardona S. M. Dijkstra I. M. (2006). Control of microglial neurotoxicity by the fractalkine receptor. *Nat. Neurosci.* 9 917–924. 10.1038/nn1715 16732273

[B27] Carroll J. C. Rosario E. R. Kreimer S. Villamagna A. Gentzschein E. Stanczyk F. Z. (2010). Sex differences in β-amyloid accumulation in 3xTg-AD mice: role of neonatal sex steroid hormone exposure. *Brain Res.* 1366 233–245. 10.1016/j.brainres.2010.10.009 20934413 PMC2993873

[B28] Castellano J. M. Kim J. Stewart F. R. Jiang H. DeMattos R. B. Patterson B. W. (2011). Human apoE isoforms differentially regulate brain amyloid- peptide clearance. *Sci. Transl. Med.* 3:89ra57. 10.1126/scitranslmed.3002156 21715678 PMC3192364

[B29] Chandrasekaran A. Avci H. X. Leist M. Kobolak J. Dinnyes A. (2016). Astrocyte differentiation of human pluripotent stem cells: new tools for neurological disorder research. *Front. Cell. Neurosci.* 10:215. 10.3389/fncel.2016.00215 27725795 PMC5035736

[B30] Charriaut-Marlangue C. Leconte C. Csaba Z. Chafa L. Pansiot J. Talatizi M. (2018). Sex differences in the effects of PARP inhibition on microglial phenotypes following neonatal stroke. *Brain. Behav. Immun.* 73 375–389. 10.1016/j.bbi.2018.05.022 29852289

[B31] Cho S. H. Sun B. Zhou Y. Kauppinen T. M. Halabisky B. Wes P. (2011). CX3CR1 protein signaling modulates microglial activation and protects against plaque-independent cognitive deficits in a mouse model of Alzheimer disease. *J. Biol. Chem.* 286 32713–32722. 10.1074/jbc.M111.254268 21771791 PMC3173153

[B32] Clinton L. K. Billings L. M. Green K. N. Caccamo A. Ngo J. Oddo S. (2007). Age-dependent sexual dimorphism in cognition and stress response in the 3xTg-AD mice. *Neurobiol. Dis.* 28 76–82. 10.1016/j.nbd.2007.06.013 17659878 PMC2756084

[B33] Combs C. K. Johnson D. E. Cannady S. B. Lehman T. M. Landreth G. E. (1999). Identification of microglial signal transduction pathways mediating a neurotoxic response to amyloidogenic fragments of β-amyloid and prion proteins. *J. Neurosci.* 19 928–939. 10.1523/JNEUROSCI.19-03-00928.1999 9920656 PMC6782151

[B34] Corder E. H. Saunders A. M. Strittmatter W. J. Schmechel D. E. Gaskell P. C. Small G. W. (1993). Gene dose of apolipoprotein E type 4 allele and the risk of Alzheimer’s disease in late onset families. *Science* 261 921–923. 8346443 10.1126/science.8346443

[B35] Czirr E. Castello N. A. Mosher K. I. Castellano J. M. Hinkson I. V. Lucin K. M. (2017). Microglial complement receptor 3 regulates brain Aβ levels through secreted proteolytic activity. *J. Exp. Med.* 214 1081–1092. 10.1084/jem.20162011 28298456 PMC5379986

[B36] Daborg J. Andreasson U. Pekna M. Lautner R. Hanse E. Minthon L. (2012). Cerebrospinal fluid levels of complement proteins C3, C4 and CR1 in Alzheimer’s disease. *J. Neural Transm.* 119 789–797. 10.1007/s00702-012-0797-8 22488444

[B37] D’Amelio M. Cavallucci V. Middei S. Marchetti C. Pacioni S. Ferri A. (2011). Caspase-3 triggers early synaptic dysfunction in a mouse model of Alzheimer’s disease. *Nat. Neurosci.* 14 69–79. 10.1038/nn.2709 21151119

[B38] Davis M. M. (2012). Immunology taught by humans. *Sci. Transl. Med.* 4:117fs2. 10.1126/scitranslmed.3003385 22261029 PMC3762495

[B39] DeMattos R. B. Cirrito J. R. Parsadanian M. May P. C. O’Dell M. A. Taylor J. W. (2004). ApoE and clusterin cooperatively suppress Aβ levels and deposition: evidence that ApoE regulates extracellular Aβ metabolism *in vivo*. *Neuron* 41 193–202. 10.1016/S0896-6273(03)00850-X 14741101

[B40] Dionisio-Santos D. A. Olschowka J. A. O’Banion M. K. (2019). Exploiting microglial and peripheral immune cell crosstalk to treat Alzheimer’s disease. *J. Neuroinflammation* 16:74. 10.1186/s12974-019-1453-0 30953557 PMC6449993

[B41] Douvaras P. Sun B. Wang M. Kruglikov I. Lallos G. Zimmer M. (2017). Directed differentiation of human pluripotent stem cells to microglia. *Stem Cell Rep.* 8 1516–1524. 10.1016/j.stemcr.2017.04.023 28528700 PMC5470097

[B42] Dubbelaar M. L. Kracht L. Eggen B. J. L. Boddeke E. W. G. M. (2018). The Kaleidoscope of microglial phenotypes. *Front. Immunol.* 9:1753. 10.3389/fimmu.2018.01753 30108586 PMC6079257

[B43] Dumont M. Beal M. F. (2011). Neuroprotective strategies involving ROS in Alzheimer disease. *Free Radic. Biol. Med.* 51 1014–1026. 10.1016/j.freeradbiomed.2010.11.026 21130159 PMC3070183

[B44] Egensperger R. Kösel S. Eitzen U. Graeber M. B. (1998). Microglial activation in Alzheimer disease: association with APOE genotype. *Brain Pathol.* 8 439–447. 10.1111/j.1750-3639.1998.tb00166.x 9669695 PMC8098510

[B45] Erb L. Woods L. T. Khalafalla M. G. Weisman G. A. (2018). Purinergic signaling in Alzheimer’s disease. *Brain Res. Bull.* 10.1016/j.brainresbull.2018.10.014 [Epub ahead of print]. 30472151

[B46] Fagan A. M. Watson M. Parsadanian M. Bales K. R. Paul S. M. Holtzman D. M. (2002). Human and murine ApoE markedly alters Aβ metabolism before and after plaque formation in a mouse model of Alzheimer’s disease. *Neurobiol. Dis.* 9 305–318. 10.1006/nbdi.2002.0483 11950276

[B47] Fan Z. Brooks D. J. Okello A. Edison P. (2017). An early and late peak in microglial activation in Alzheimer’s disease trajectory. *Brain* 140 792–803. 10.1093/brain/aww349 28122877 PMC5837520

[B48] Färber K. Kettenmann H. (2006). Functional role of calcium signals for microglial function. *Glia* 54 656–665. 10.1002/glia.20412 17006894

[B49] Ferretti M. T. Iulita M. F. Cavedo E. Chiesa P. A. Schumacher Dimech A. Santuccione Chadha A. (2018). Sex differences in Alzheimer disease - the gateway to precision medicine. *Nat. Rev. Neurol.* 14 457–469. 10.1038/s41582-018-0032-9 29985474

[B50] Fonseca M. I. Zhou J. Botto M. Tenner A. J. (2004). Absence of C1q leads to less neuropathology in transgenic mouse models of Alzheimer’s disease. *J. Neurosci.* 24 6457–6465. 10.1523/jneurosci.0901-04.2004 15269255 PMC6729885

[B51] Frank S. Burbach G. J. Bonin M. Walter M. Streit W. Bechmann I. (2008). TREM2 is upregulated in amyloid plaque-associated microglia in aged APP23 transgenic mice. *Glia* 56 1438–1447. 10.1002/glia.20710 18551625

[B52] Friedman B. A. Srinivasan K. Ayalon G. Meilandt W. J. Lin H. Huntley M. A. (2018). Diverse brain myeloid expression profiles reveal distinct microglial activation states and aspects of Alzheimer’s disease not evident in mouse models. *Cell Rep.* 22 832–847. 10.1016/j.celrep.2017.12.066 29346778

[B53] Fu H. Liu B. Frost J. L. Hong S. Jin M. Ostaszewski B. (2012). Complement component C3 and complement receptor type 3 contribute to the phagocytosis and clearance of fibrillar Aβ by microglia. *Glia* 60 993–1003. 10.1002/glia.22331 22438044 PMC3325361

[B54] Fuhrmann M. Bittner T. Jung C. K. E. Burgold S. Page R. M. Mitteregger G. (2010). Microglial Cx3cr1 knockout prevents neuron loss in a mouse model of Alzheimer’s disease. *Nat. Neurosci.* 13 411–413. 10.1038/nn.2511 20305648 PMC4072212

[B55] Galatro T. F. Holtman I. R. Lerario A. M. Vainchtein I. D. Brouwer N. Sola P. R. (2017). Transcriptomic analysis of purified human cortical microglia reveals age-associated changes. *Nat Neurosci.* 20 1162–1171. 10.1038/nn.4597 28671693

[B56] Gallagher J. J. Minogue A. M. Lynch M. A. (2013). Impaired performance of female APP/PS1 mice in the morris water maze is coupled with increased Aβ accumulation and microglial activation. *Neurodegener. Dis.* 11 33–41. 10.1159/000337458 22627185

[B57] Garcia-Reitboeck P. Phillips A. Piers T. M. Villegas-Llerena C. Butler M. Mallach A. (2018). Human induced pluripotent stem cell-derived microglia-like cells harboring TREM2 missense mutations show specific deficits in phagocytosis. *Cell Rep.* 24 2300–2311. 10.1016/j.celrep.2018.07.094 30157425 PMC6130048

[B58] Ginhoux F. Greter M. Leboeuf M. Nandi S. See P. Gokhan S. (2010). Fate mapping analysis reveals that adult microglia derive from primitive macrophages. *Science* 330 841–845. 10.1126/science.1194637 20966214 PMC3719181

[B59] Gosselin D. Skola D. Coufal N. G. Holtman I. R. Schlachetzki J. C. M. Sajti E. (2017). An environment-dependent transcriptional network specifies human microglia identity. *Science* 356:eaal3222. 10.1126/science.aal3222 28546318 PMC5858585

[B60] Grabert K. Michoel T. Karavolos M. H. Clohisey S. Baillie J. K. Stevens M. P. (2016). Microglial brain region-dependent diversity and selective regional sensitivities to aging. *Nat. Neurosci.* 19 504–516. 10.1038/nn.4222 26780511 PMC4768346

[B61] Graeber M. B. Kösel S. Egensperger R. Banati R. B. Müller U. Bise K. (1997). Rediscovery of the case described by Alois Alzheimer in 1911: historical, histological and molecular genetic analysis. *Neurogenetics* 1 73–80. 10.1007/s100480050011 10735278

[B62] Griffin W. S. Stanley L. C. Ling C. White L. MacLeod V. Perrot L. J. (1989). Brain interleukin 1 and S-100 immunoreactivity are elevated in Down syndrome and Alzheimer disease. *Proc. Natl. Acad. Sci.U.S.A.* 86 7611–7615. 10.1073/pnas.86.19.7611 2529544 PMC298116

[B63] Guerreiro R. Wojtas A. Bras J. Carrasquillo M. Rogaeva E. Majounie E. (2013). TREM2 variants in Alzheimer’s disease. *N. Engl. J. Med.* 368 117–127. 10.1056/NEJMoa1211851 23150934 PMC3631573

[B64] Guneykaya D. Ivanov A. Hernandez D. P. Haage V. Wojtas B. Meyer N. (2018). Transcriptional and translational differences of microglia from male and female brains. *Cell Rep.* 24 2773–2783.e6. 10.1016/J.CELREP.2018.08.001 30184509

[B65] Guo L. Ladu M. J. Van Eldik L. J. (2004). A dual role for apolipoprotein E in neuroinflammation anti-and pro-inflammatory activity. *J. Mol. Neurosci.* 23 205–212. 15181248 10.1385/JMN:23:3:205

[B66] Haenseler W. Sansom S. N. Buchrieser J. Newey S. E. Moore C. S. Nicholls F. J. (2017). A highly efficient human pluripotent stem cell microglia model displays a neuronal-co-culture-specific expression profile and inflammatory response. *Stem Cell Rep.* 8 1727–1742. 10.1016/j.stemcr.2017.05.017 28591653 PMC5470330

[B67] Halle A. Hornung V. Petzold G. C. Stewart C. R. Monks B. G. Reinheckel T. (2008). The NALP3 inflammasome is involved in the innate immune response to amyloid-β. *Nat. Immunol.* 9 857–865. 10.1038/ni.1636 18604209 PMC3101478

[B68] Hammond T. R. Dufort C. Dissing-Olesen L. Giera S. Young A. Wysoker A. (2019). Single-cell RNA sequencing of microglia throughout the mouse lifespan and in the injured brain reveals complex cell-state changes. *Immunity* 50 253–271.e6. 10.1016/j.immuni.2018.11.004 30471926 PMC6655561

[B69] Hanisch U. K. Ketternmann H. (2007). Microglia: active sensor and versatile effector cells in the normal and pathologic brain. *Nat. Neurosci.* 10 1387–1394. 10.1038/nn1997 17965659

[B70] Hansen D. V. Hanson J. E. Sheng M. (2018). Microglia in Alzheimer’s disease. *J. Cell Biol.* 217 459–472. 10.1083/jcb.201709069 29196460 PMC5800817

[B71] Harrigan T. J. Abdullaev I. F. Jourd’heuil D. Mongin A. A. (2008). Activation of microglia with zymosan promotes excitatory amino acid release via volume-regulated anion channels: the role of NADPH oxidases. *J. Neurochem.* 106 2449–2462. 10.1111/j.1471-4159.2008.05553.x 18624925 PMC2574595

[B72] Harvey R. J. Skelton-Robinson M. Rossor M. N. (2003). The prevalence and causes of dementia in people under the age of 65 years. *J. Neurol. Neurosurg. Psychiatry* 74 1206–1209. 10.1136/JNNP.74.9.1206 12933919 PMC1738690

[B73] Hashimoto T. Serrano-Pozo A. Hori Y. Adams K. W. Takeda S. Banerji A. O. (2012). Apolipoprotein E, especially apolipoprotein E4, increases the oligomerization of amyloid peptide. *J. Neurosci.* 32 15181–15192. 10.1523/JNEUROSCI.1542-12.2012 23100439 PMC3493562

[B74] Haynes S. E. Hollopeter G. Yang G. Kurpius D. Dailey M. E. Gan W.-B. (2006). The P2Y12 receptor regulates microglial activation by extracellular nucleotides. *Nat. Neurosci.* 9 1512–1519. 10.1038/nn1805 17115040

[B75] Hebert L. E. Weuve J. Scherr P. A. Evans D. A. (2013). Alzheimer disease in the United States (2010-2050) estimated using the 2010 census. *Neurology* 80 1778–1783. 10.1212/WNL.0b013e31828726f5 23390181 PMC3719424

[B76] Heneka M. T. Kummer M. P. Stutz A. Delekate A. Schwartz S. Vieira-Saecker A. (2013). NLRP3 is activated in Alzheimer’s disease and contributes to pathology in APP/PS1 mice. *Nature* 493 674–678. 10.1038/nature11729 23254930 PMC3812809

[B77] Heuer E. Rosen R. F. Cintron A. Walker L. C. (2012). Nonhuman primate models of Alzheimer-like cerebral proteopathy. *Curr. Pharm. Des.* 18 1159–1169. 22288403 10.2174/138161212799315885 PMC3381739

[B78] Hirbec H. E. Noristani H. N. Perrin F. E. (2017). Microglia responses in acute and chronic neurological diseases: what microglia-specific transcriptomic studies taught (and did Not Teach) Us. *Front. Aging Neurosci.* 9:227. 10.3389/fnagi.2017.00227 28785215 PMC5519576

[B79] Hoeffel G. Ginhoux F. (2015). Ontogeny of tissue-resident macrophages. *Front. Immunol.* 6:486. 10.3389/fimmu.2015.00486 26441990 PMC4585135

[B80] Hoffmann A. Kann O. Ohlemeyer C. Hanisch U.-K. Kettenmann H. (2003). Elevation of basal intracellular calcium as a central element in the activation of brain macrophages (microglia): suppression of receptor-evoked calcium signaling and control of release function. *J. Neurosci.* 23 4410–4419. 10.1523/JNEUROSCI.23-11-04410.2003 12805281 PMC6740788

[B81] Holtman I. R. Raj D. D. Miller J. A. Schaafsma W. Yin Z. Brouwer N. (2015). Induction of a common microglia gene expression signature by aging and neurodegenerative conditions: a co-expression meta-analysis. *Acta Neuropathol. Commun.* 3:31. 10.1186/s40478-015-0203-5 26001565 PMC4489356

[B82] Holtman I. R. Skola D. Glass C. K. (2017). Transcriptional control of microglia phenotypes in health and disease. *J. Clin. Invest.* 127 3220–3229. 10.1172/JCI90604 28758903 PMC5669536

[B83] Holtzman D. M. Bales K. R. Wu S. Bhat P. Parsadanian M. Fagan A. M. (1999). Expression of human apolipoprotein E reduces amyloid-β deposition in a mouse model of Alzheimer’s disease. *J. Clin. Invest.* 103 R15–R21. 10.1172/JCI6179 10079115 PMC408154

[B84] Hong S. Beja-Glasser V. F. Nfonoyim B. M. Frouin A. Li S. Ramakrishnan S. (2016). Complement and microglia mediate early synapse loss in Alzheimer mouse models. *Science* 352 712–716. 10.1126/science.aad8373 27033548 PMC5094372

[B85] Hoshiko M. Arnoux I. Avignone E. Yamamoto N. Audinat E. (2012). Deficiency of the microglial receptor CX3CR1 impairs postnatal functional development of thalamocortical synapses in the barrel cortex. *J. Neurosci.* 32 15106–15111. 10.1523/JNEUROSCI.1167-12.2012 23100431 PMC6704837

[B86] Hudry E. Dashkoff J. Roe A. D. Takeda S. Koffie R. M. Hashimoto T. (2013). Gene transfer of human Apoe isoforms results in differential modulation of amyloid deposition and neurotoxicity in mouse brain. *Sci. Transl. Med.* 5:212ra161. 10.1126/scitranslmed.3007000 24259049 PMC4334150

[B87] Janus C. Flores A. Y. Xu G. Borchelt D. R. (2015). Behavioral abnormalities in APP_Swe_/PS1dE9 mouse model of AD-like pathology: comparative analysis across multiple behavioral domains. *Neurobiol. Aging* 36 2519–2532. 10.1016/j.neurobiolaging.2015.05.010 26089165

[B88] Jay T. R. Miller C. M. Cheng P. J. Graham L. C. Bemiller S. Broihier M. L. (2015). TREM2 deficiency eliminates TREM2+ inflammatory macrophages and ameliorates pathology in Alzheimer’s disease mouse models. *J. Exp. Med.* 212 287–295. 10.1084/jem.20142322 25732305 PMC4354365

[B89] Jiang T. Tan L. Zhu X.-C. Zhang Q.-Q. Cao L. Tan M.-S. (2014). Upregulation of TREM2 ameliorates neuropathology and rescues spatial cognitive impairment in a transgenic mouse model of Alzheimer’s disease. *Neuropsychopharmacology* 39 2949–2962. 10.1038/npp.2014.164 25047746 PMC4229581

[B90] Jiao S.-S. Bu X.-L. Liu Y.-H. Zhu C. Wang Q.-H. Shen L.-L. (2016). Sex dimorphism profile of Alzheimer’s disease-type pathologies in an APP/PS1 mouse model. *Neurotox. Res.* 29 256–266. 10.1007/s12640-015-9589-x 26707129

[B91] Jin S. C. Benitez B. A. Karch C. M. Cooper B. Skorupa T. Carrell D. (2014). Coding variants in TREM2 increase risk for Alzheimer’s disease. *Hum. Mol. Genet.* 23 5838–5846. 10.1093/hmg/ddu277 24899047 PMC4189899

[B92] Jonsson T. Stefansson H. Steinberg S. Jonsdottir I. Jonsson P. V. Snaedal J. (2013). Variant of TREM2 associated with the risk of Alzheimer’s disease. *N. Engl. J. Med.* 368 107–116. 10.1056/NEJMoa1211103 23150908 PMC3677583

[B93] Joshi P. Turola E. Ruiz A. Bergami A. Libera D. D. Benussi L. (2014). Microglia convert aggregated amyloid-β into neurotoxic forms through the shedding of microvesicles. *Cell Death Differ.* 21 582–593. 10.1038/cdd.2013.180 24336048 PMC3950321

[B94] Jullienne A. Salehi A. Affeldt B. Baghchechi M. Haddad E. Avitua A. (2018). Male and female mice exhibit divergent responses of the cortical vasculature to traumatic brain injury. *J. Neurotrauma* 35 1646–1658. 10.1089/neu.2017.5547 29648973 PMC6016102

[B95] Keren-Shaul H. Spinrad A. Weiner A. Matcovitch-Natan O. Dvir-Szternfeld R. Ulland T. K. (2017). A unique microglia type associated with restricting development of Alzheimer’s disease. *Cell* 169 1276–1290.e17. 10.1016/j.cell.2017.05.018 28602351

[B96] Kierdorf K. Prinz M. (2017). Microglia in steady state. *J. Clin. Invest.* 127 3201–3209. 10.1172/JCI90602 28714861 PMC5669563

[B97] Kiialainen A. Hovanes K. Paloneva J. Kopra O. Peltonen L. (2005). Dap12 and Trem2, molecules involved in innate immunity and neurodegeneration, are co-expressed in the CNS. *Neurobiol. Dis.* 18 314–322. 10.1016/j.nbd.2004.09.007 15686960

[B98] Kim H. J. Ajit D. Peterson T. S. Wang Y. Camden J. M. Gibson Wood W. (2012). Nucleotides released from Aβ1-42-treated microglial cells increase cell migration and Aβ1-42 uptake through P2Y2 receptor activation. *J. Neurochem.* 121 228–238. 10.1111/j.1471-4159.2012.07700.x 22353164 PMC3323761

[B99] Kim T. S. Lim H. K. Lee J. Y. Kim D. J. Park S. Lee C. (2008). Changes in the levels of plasma soluble fractalkine in patients with mild cognitive impairment and Alzheimer’s disease. *Neurosci. Lett.* 436 196–200. 10.1016/j.neulet.2008.03.019 18378084

[B100] Koffie R. M. Hashimoto T. Tai H.-C. Kay K. R. Serrano-Pozo A. Joyner D. (2012). Apolipoprotein E4 effects in Alzheimer’s disease are mediated by synaptotoxic oligomeric amyloid-β. *Brain* 135 2155–2168. 10.1093/brain/aws127 22637583 PMC3381721

[B101] Krabbe G. Halle A. Matyash V. Rinnenthal J. L. Eom G. D. Bernhardt U. (2013). Functional impairment of microglia coincides with beta-amyloid deposition in mice with alzheimer-like pathology. *PLoS One* 8:e60921. 10.1371/journal.pone.0060921 23577177 PMC3620049

[B102] Krasemann S. Madore C. Cialic R. Baufeld C. Calcagno N. El Fatimy R. (2017). The TREM2-APOE pathway drives the transcriptional phenotype of dysfunctional microglia in neurodegenerative diseases. *Immunity* 47 566–581.e9. 10.1016/j.immuni.2017.08.008 28930663 PMC5719893

[B103] Labzin L. I. Heneka M. T. Latz E. (2017). Innate Immunity and Neurodegeneration. *Annu. Rev. Med.* 69 437–449. 10.1146/annurev-med-050715-104343 29106805

[B104] Lai M. K. P. Tan M. G. K. Kirvell S. Hobbs C. Lee J. Esiri M. M. (2008). Selective loss of P2Y2 nucleotide receptor immunoreactivity is associated with Alzheimer’s disease neuropathology. *J. Neural Transm.* 115 1165–1172. 10.1007/s00702-008-0067-y 18506388

[B105] Lajaunias F. Dayer J. M. Chizzolini C. (2005). Constitutive repressor activity of CD33 on human monocytes requires sialic acid recognition and phosphoinositide 3-kinase-mediated intracellular signaling. *Eur. J. Immunol.* 35 243–251. 10.1002/eji.200425273 15597323

[B106] Le Corre M. Noristani H. N. Mestre-Frances N. Saint-Martin G. P. Coillot C. Goze-Bac C. (2018). A Novel Translational Model of Spinal Cord Injury in Nonhuman Primate. *Neurotherapeutics* 15 751–769. 10.1007/s13311-017-0589-9 29181770 PMC6095780

[B107] Lee C. Y. D. Daggett A. Gu X. Jiang L.-L. Langfelder P. Li X. (2018). Elevated TREM2 gene dosage reprograms microglia responsivity and ameliorates pathological phenotypes in Alzheimer’s disease models. *Neuron* 97 1032–1048.e5. 10.1016/j.neuron.2018.02.002 29518357 PMC5927822

[B108] Lee C. Z. W. Kozaki T. Ginhoux F. (2018). Studying tissue macrophages in vitro: are iPSC-derived cells the answer? *Nat. Rev. Immunol.* 18 716–725. 10.1038/s41577-018-0054-y 30140052

[B109] Lee J. H. Yu W. H. Kumar A. Lee S. Mohan P. S. Peterhoff C. M. (2010). Lysosomal proteolysis and autophagy require presenilin 1 and are disrupted by Alzheimer-related PS1 mutations. *Cell* 141 1146–1158. 10.1016/j.cell.2010.05.008 20541250 PMC3647462

[B110] Lian H. Litvinchuk A. Chiang A. C.-A. Aithmitti N. Jankowsky J. L. Zheng H. (2016). Astrocyte-microglia cross talk through complement activation modulates amyloid pathology in mouse models of Alzheimer’s disease. *J. Neurosci.* 36 577–589. 10.1523/jneurosci.2117-15.2016 26758846 PMC4710776

[B111] Liddelow S. A. Barres B. A. (2017). Reactive astrocytes: production, function, and therapeutic potential. *Immunity* 46 957–967. 10.1016/j.immuni.2017.06.006 28636962

[B112] Loerch P. M. Lu T. Dakin K. A. Vann J. M. Isaacs A. Geula C. (2008). Evolution of the aging brain transcriptome and synaptic regulation. *PLoS One* 3:e3329. 10.1371/journal.pone.0003329 18830410 PMC2553198

[B113] Lynch J. R. Morgan D. Mance J. Matthew W. D. Laskowitz D. T. (2001). Apolipoprotein E modulates glial activation and the endogenous central nervous system inflammatory response. *J. Neuroimmunol.* 114 107–113. 10.1016/S0165-5728(00)00459-8 11240021

[B114] Lyons A. Lynch A. M. Downer E. J. Hanley R. O’Sullivan J. B. Smith A. (2009). Fractalkine-induced activation of the phosphatidylinositol-3 kinase pathway attentuates microglial activation *in vivo* and *in vitro*. *J. Neurochem.* 110 1547–1556. 10.1111/j.1471-4159.2009.06253.x 19627440

[B115] Macosko E. Z. Basu A. Satija R. Nemesh J. Shekhar K. Goldman M. (2015). Highly parallel genome-wide expression profiling of individual cells using nanoliter droplets. *Cell* 161 1202–1214. 10.1016/j.cell.2015.05.002 26000488 PMC4481139

[B116] Maier M. Peng Y. Jiang L. Seabrook T. J. Carroll M. C. Lemere C. A. (2008). Complement C3 deficiency leads to accelerated amyloid plaque deposition and neurodegeneration and modulation of the microglia/macrophage phenotype in amyloid precursor protein transgenic mice. *J. Neurosci.* 28 6333–6341. 10.1523/jneurosci.0829-08.2008 18562603 PMC3329761

[B117] Mapplebeck J. C. S. Dalgarno R. Tu Y. Moriarty O. Beggs S. Kwok C. H. T. (2018). Microglial P2X4R-evoked pain hypersensitivity is sexually dimorphic in rats. *Pain* 159 1752–1763. 10.1097/j.pain.0000000000001265 29927790

[B118] Martin E. Amar M. Dalle C. Youssef I. Boucher C. Le Duigou C. (2018). New role of P2X7 receptor in an Alzheimer’s disease mouse model. *Mol. Psychiatry* 24 108–125. 10.1038/s41380-018-0108-3 29934546 PMC6756107

[B119] Martinon F. Burns K. Tschopp J. (2002). The Inflammasome: a molecular platform triggering activation of inflammatory caspases and processing of proIL-β. *Mol. Cell* 10 417–426. 10.1016/S1097-2765(02)00599-3 12191486

[B120] Masuda T. Sankowski R. Staszewski O. Bottcher C. Amann L. Scheiwe C. (2019). Spatial and temporal heterogeneity of mouse and human microglia at single-cell resolution. *Nature* 566 388–392. 10.1038/s41586-019-0924-x 30760929

[B121] McLarnon J. G. (2005). Purinergic mediated changes in Ca2+ mobilization and functional responses in microglia: effects of low levels of ATP. *J. Neurosci. Res.* 81 349–356. 10.1002/jnr.20475 15948175

[B122] McLarnon J. G. Choi H. B. Lue L.-F. Walker D. G. Kim S. U. (2005). Perturbations in calcium-mediated signal transduction in microglia from Alzheimer’s disease patients. *J. Neurosci. Res.* 81 426–435. 10.1002/jnr.20487 15948178

[B123] McLarnon J. G. Ryu J. K. Walker D. G. Choi H. B. (2006). Upregulated expression of purinergic P2X 7 receptor in Alzheimer disease and amyloid-β peptide-treated microglia and in peptide-injected rat hippocampus. *J. Neuropathol. Exp. Neurol.* 65 1090–1097. 10.1097/01.jnen.0000240470.97295.d3 17086106

[B124] McQuade A. Blurton-Jones M. (2019). Microglia in Alzheimer’s disease: exploring how genetics and phenotype influence risk. *J. Mol. Biol.* 431 1805–1817. 10.1016/J.JMB.2019.01.045 30738892 PMC6475606

[B125] Melief J. Koning N. Schuurman K. G. Van De Garde M. D. B. Smolders J. Hoek R. M. (2012). Phenotyping primary human microglia: tight regulation of LPS responsiveness. *Glia* 60 1506–1517. 10.1002/glia.22370 22740309

[B126] Mestre-Francés N. Keller E. Calenda A. Barelli H. Checler F. Bons N. (2000). Immunohistochemical analysis of cerebral cortical and vascular lesions in the primate *Microcebus murinus* reveal distinct amyloid beta1-42 and beta1-40 immunoreactivity profiles. *Neurobiol. Dis.* 7 1–8. 10.1006/nbdi.1999.0270 10671318

[B127] Mildner A. Huang H. Radke J. Stenzel W. Priller J. (2017). P2Y 12 receptor is expressed on human microglia under physiological conditions throughout development and is sensitive to neuroinflammatory diseases. *Glia* 65 375–387. 10.1002/glia.23097 27862351

[B128] Minett T. Classey J. Matthews F. E. Fahrenhold M. Taga M. Brayne C. (2016). Microglial immunophenotype in dementia with Alzheimer’s pathology. *J. Neuroinflammation* 13:135. 10.1186/s12974-016-0601-z 27256292 PMC4890505

[B129] Muffat J. Li Y. Yuan B. Mitalipova M. Omer A. Corcoran S. (2016). Efficient derivation of microglia-like cells from human pluripotent stem cells. *Nat. Med.* 22 1358–1367. 10.1038/nm.4189 27668937 PMC5101156

[B130] Naslund J. Thyberg J. Wernstedt C. Ft Karlstrom A. Bogdanovic N. Samuel Gandy I. E. (1995). Characterization of stable complexes involving apolipoprotein E and the amyloid B peptide in Alzheimer’s disease brain. *Neuron* 15 219–228. 7619525 10.1016/0896-6273(95)90079-9

[B131] Nelson L. H. Warden S. Lenz K. M. (2017). Sex differences in microglial phagocytosis in the neonatal hippocampus. *Brain. Behav. Immun.* 64 11–22. 10.1016/j.bbi.2017.03.010 28341582 PMC5512447

[B132] Ni J. Wang P. Zhang J. Chen W. Gu L. (2013). Silencing of the P2X7 receptor enhances amyloid-β phagocytosis by microglia. *Biochem. Biophys. Res. Commun.* 434 363–369. 10.1016/j.bbrc.2013.03.079 23562658

[B133] Nissen J. C. (2017). Microglial function across the spectrum of age and gender. *Int. J. Mol. Sci.* 18:E561. 10.3390/ijms18030561 28273860 PMC5372577

[B134] Nixon R. A. Wegiel J. Kumar A. Yu W. H. Peterhoff C. Cataldo A. (2005). Extensive involvement of autophagy in Alzheimer disease: an immuno-electron microscopy study. *J. Neuropathol. Exp. Neurol.* 64 113–122. 10.1093/jnen/64.2.113 15751225

[B135] Nizami S. Hall-Roberts H. Warrier S. Cowley S. A. Di Daniel E. (2019). Microglial inflammation and phagocytosis in Alzheimer’s disease: potential therapeutic targets. *Br. J. Pharmacol.* 10.1111/bph.14618 [Epub ahead of print]. 30740661 PMC6715590

[B136] Nnah I. C. Wessling-Resnick M. (2018). Brain iron homeostasis: a focus on microglial iron. *Pharmaceuticals* 11:E129. 10.3390/ph11040129 30477086 PMC6316365

[B137] Orre M. Kamphuis W. Osborn L. M. Jansen A. H. Kooijman L. Bossers K. (2014). Isolation of glia from Alzheimer’s mice reveals inflammation and dysfunction. *Neurobiol. Aging* 35 2746–2760. 10.1016/j.neurobiolaging.2014.06.004 25002035

[B138] Pandya H. Shen M. J. Ichikawa D. M. Sedlock A. B. Choi Y. Johnson K. R. (2017). Differentiation of human and murine induced pluripotent stem cells to microglia-like cells. *Nat. Neurosci.* 20 753–759. 10.1038/nn.4534 28253233 PMC5404968

[B139] Paolicelli R. C. Bolasco G. Pagani F. Maggi L. Scianni M. Panzanelli P. (2011). Synaptic pruning by microglia is necessary for normal brain development. *Science* 333 1456–1458. 10.1126/science.1202529 21778362

[B140] Parhizkar S. Arzberger T. Brendel M. Kleinberger G. Deussing M. Focke C. (2019). Loss of TREM2 function increases amyloid seeding but reduces plaque-associated ApoE. *Nat. Neurosci.* 22 191–204. 10.1038/s41593-018-0296-9 30617257 PMC6417433

[B141] Perkins A. E. Piazza M. K. Deak T. (2018). Stereological analysis of microglia in aged male and female fischer 344 rats in socially relevant brain regions. *Neuroscience* 377 40–52. 10.1016/j.neuroscience.2018.02.028 29496632 PMC5882587

[B142] Piccio L. Buonsanti C. Mariani M. Cella M. Gilfillan S. Cross A. H. (2007). Blockade of TREM-2 exacerbates experimental autoimmune encephalomyelitis. *Eur. J. Immunol.* 37 1290–1301. 10.1002/eji.200636837 17407101

[B143] Pocock J. M. Piers T. M. (2018). Modelling microglial function with induced pluripotent stem cells: an update. *Nat. Rev. Neurosci.* 19 445–452. 10.1038/s41583-018-0030-3 29977068

[B144] Poon A. Zhang Y. Chandrasekaran A. Phanthong P. Schmid B. Nielsen T. T. (2017). Modeling neurodegenerative diseases with patient-derived induced pluripotent cells: possibilities and challenges. *N. Biotechnol.* 39 190–198. 10.1016/j.nbt.2017.05.009 28579476

[B145] Próchnicki T. Mangan M. S. Latz E. (2016). Recent insights into the molecular mechanisms of the NLRP3 inflammasome activation. *F1000Research* 5:1469. 10.12688/f1000research.8614.1 27508077 PMC4963208

[B146] Ransohoff R. M. (2018). All (animal) models (of neurodegeneration) are wrong. Are they also useful? *J. Exp. Med.* 215 2955–2958. 10.1084/jem.20182042 30459159 PMC6279414

[B147] Ransohoff R. M. Perry V. H. (2009). Microglial physiology: unique stimuli, specialized responses. *Annu. Rev. Immunol.* 27 119–145. 10.1146/annurev.immunol.021908.132528 19302036

[B148] Rodriguez G. A. Tai L. M. LaDu M. J. Rebeck G. W. (2014). Human APOE4 increases microglia reactivity at Aβ plaques in a mouse model of Aβ deposition. *J. Neuroinflammation* 11:111. 10.1186/1742-2094-11-111 24948358 PMC4077554

[B149] Saito T. Matsuba Y. Mihira N. Takano J. Nilsson P. Itohara S. (2014). Single App knock-in mouse models of Alzheimer’s disease. *Nat. Neurosci.* 17 661–663. 10.1038/nn.3697 24728269

[B150] Sakakibara Y. Sekiya M. Saito T. Saido T. C. Iijima K. M. (2018). Cognitive and emotional alterations in App knock-in mouse models of Aβ amyloidosis. *BMC Neurosci.* 19:46. 10.1186/s12868-018-0446-8 30055565 PMC6064053

[B151] Sala Frigerio C. Wolfs L. Fattorelli N. Thrupp N. Voytyuk I. Schmidt I. (2019). The major risk factors for Alzheimer’s disease: age, sex, and genes modulate the microglia response to Aβ plaques. *Cell Rep.* 27 1293–1306.e6. 10.1016/j.celrep.2019.03.099 31018141 PMC7340153

[B152] Salamanca L. Mechawar N. Murai K. K. Balling R. Bouvier D. S. Skupin A. (2019). MIC-MAC: An automated pipeline for high-throughput characterization and classification of three-dimensional microglia morphologies in mouse and human postmortem brain samples. *Glia* 67 1496–1509. 10.1002/glia.23623 30983036 PMC6617786

[B153] Saman S. Kim W. Raya M. Visnick Y. Miro S. Saman S. (2012). Exosome-associated tau is secreted in tauopathy models and is selectively phosphorylated in cerebrospinal fluid in early Alzheimer disease. *J. Biol. Chem.* 287 3842–3849. 10.1074/jbc.M111.277061 22057275 PMC3281682

[B154] Sanchez-Mejias E. Navarro V. Jimenez S. Sanchez-Mico M. Sanchez-Varo R. Nuñez-Diaz C. (2016). Soluble phospho-tau from Alzheimer’s disease hippocampus drives microglial degeneration. *Acta Neuropathol.* 132 897–916. 10.1007/s00401-016-1630-5 27743026 PMC5106501

[B155] Sanz J. M. Falzoni S. Rizzo R. Cipollone F. Zuliani G. Di Virgilio F. (2014). Possible protective role of the 489C&gt;T P2X7R polymorphism in Alzheimer’s disease. *Exp. Gerontol.* 60 117–119. 10.1016/j.exger.2014.10.009 25456845 PMC4266448

[B156] Sarasa M. Pesini P. (2009). Natural non-trasgenic animal models for research in Alzheimer’s disease. *Curr. Alzheimer Res.* 6 171–178. 10.2174/156720509787602834 19355852 PMC2825666

[B157] Sasaguri H. Nilsson P. Hashimoto S. Nagata K. Saito T. De Strooper B. (2017). APP mouse models for Alzheimer’s disease preclinical studies. *EMBO J.* 36 2473–2487. 10.15252/embj.201797397 28768718 PMC5579350

[B158] Schafer D. P. Lehrman E. K. Kautzman A. G. Koyama R. Mardinly A. R. Yamasaki R. (2012). Microglia sculpt postnatal neural circuits in an activity and complement-dependent manner. *Neuron* 74 691–705. 10.1016/j.neuron.2012.03.026 22632727 PMC3528177

[B159] Schmechel D. E. Saunders A. M. Strittmatter W. J. Crain B. J. Hulette C. M. Joo S. H. (1993). Increased amyloid beta-peptide deposition in cerebral cortex as a consequence of apolipoprotein E genotype in late-onset Alzheimer disease. *Proc. Natl. Acad. Sci. U.S.A.* 90 9649–9653. 8415756 10.1073/pnas.90.20.9649 PMC47627

[B160] Schmid C. D. Sautkulis L. N. Danielson P. E. Cooper J. Hasel K. W. Hilbush B. S. (2002). Heterogeneous expression of the triggering receptor expressed on myeloid cells-2 on adult murine microglia. *J. Neurochem.* 83 1309–1320. 10.1046/j.1471-4159.2002.01243.x 12472885 PMC2637869

[B161] Schmidt F. Boltze J. Jäger C. Hofmann S. Willems N. Seeger J. (2015). Detection and quantification of β-amyloid, pyroglutamyl Aβ, and tau in aged canines. *J. Neuropathol. Exp. Neurol.* 74 912–923. 10.1097/NEN.0000000000000230 26247394

[B162] Schwarz J. M. Sholar P. W. Bilbo S. D. (2012). Sex differences in microglial colonization of the developing rat brain. *J. Neurochem.* 120 948–963. 10.1111/j.1471-4159.2011.07630.x 22182318 PMC3296888

[B163] Seok J. Cuenca H. S. W. A. G. Mindrinos M. N. Bakerc H. V. Xua W. Richardsd D. R. (2013). Genomic responses in mouse models poorly mimic human inflammatory diseases. *Proc. Natl. Acad. Sci. U.S.A.* 110 3507–3512. 10.1073/pnas.1222878110 23401516 PMC3587220

[B164] Serrano-Pozo A. Qian J. Monsell S. E. Betensky R. A. Hyman B. T. Massachusetts Alzheimer P. (2015). APOEε2 is associated with milder clinical and pathological Alzheimer’s disease. *Ann. Neurol.* 77 917–929. 10.1002/ana.24369 25623662 PMC4447539

[B165] Sieber M. W. Jaenisch N. Brehm M. Guenther M. Linnartz-Gerlach B. Neumann H. (2013). Attenuated inflammatory response in triggering receptor expressed on myeloid cells 2 (TREM2) knock-out mice following stroke. *PLoS One* 8:e52982. 10.1371/journal.pone.0052982 23301011 PMC3536811

[B166] Sierra A. de Castro F. del Río-Hortega J. Rafael Iglesias-Rozas J. Garrosa M. Kettenmann H. (2016). The “Big-Bang” for modern glial biology: translation and comments on Pío del Río-Hortega 1919 series of papers on microglia. *Glia* 64 1801–1840. 10.1002/glia.23046 27634048

[B167] Smith A. M. Dragunow M. (2014). The human side of microglia. *Trends Neurosci.* 37 125–135. 10.1016/j.tins.2013.12.001 24388427

[B168] Smith A. M. Gibbons H. M. Oldfield R. L. Bergin P. M. Mee E. W. Curtis M. A. (2013). M-CSF increases proliferation and phagocytosis while modulating receptor and transcription factor expression in adult human microglia. *J. Neuroinflammation* 10:85. 10.1186/1742-2094-10-85 23866312 PMC3729740

[B169] Sorge R. E. Mapplebeck J. C. S. Rosen S. Beggs S. Taves S. Alexander J. K. (2015). Different immune cells mediate mechanical pain hypersensitivity in male and female mice. *Nat. Neurosci.* 18 1081–1085. 10.1038/nn.4053 26120961 PMC4772157

[B170] Sousa C. Biber K. Michelucci A. (2017). Cellular and molecular characterization of microglia: a unique immune cell population. *Front. Immunol.* 8:198. 10.3389/fimmu.2017.00198 28303137 PMC5332364

[B171] Stephan A. H. Barres B. A. Stevens B. (2012). The complement system: an unexpected role in synaptic pruning during development and disease. *Annu. Rev. Neurosci.* 35 369–389. 10.1146/annurev-neuro-061010-113810 22715882

[B172] Strittmatter W. J. Weisgraber K. H. Huang D. Y. Dong L.-M. Salvesen G. S. Pericak-Vance M. (1993). Binding of human apolipoprotein E to synthetic amyloid, B peptide: isoform-specific effects and implications for late-onset Alzheimer disease. *Proc. Natl. Acad. Sci. U.S.A.* 90 8098–8102. 8367470 10.1073/pnas.90.17.8098 PMC47295

[B173] Sullivan S. E. Young-Pearse T. L. (2017). Induced pluripotent stem cells as a discovery tool for Alzheimer×s disease. *Brain Res.* 1656 98–106. 10.1016/j.brainres.2015.10.005 26459988 PMC4833689

[B174] Suurväli J. Boudinot P. Kanellopoulos J. Rüütel Boudinot S. (2017). P2X4: a fast and sensitive purinergic receptor. *Biomed. J.* 40 245–256. 10.1016/j.bj.2017.06.010 29179879 PMC6138603

[B175] Takahashi K. Rochford C. D. P. Neumann H. (2005). Clearance of apoptotic neurons without inflammation by microglial triggering receptor expressed on myeloid cells-2. *J. Exp. Med.* 201 647–657. 10.1084/jem.20041611 15728241 PMC2213053

[B176] Takata K. Kozaki T. Lee C. Z. W. Thion M. S. Otsuka M. Lim S. (2017). Induced-pluripotent-stem-cell-derived primitive macrophages provide a platform for modeling tissue-resident macrophage differentiation and function. *Immunity* 47 183–198.e6. 10.1016/j.immuni.2017.06.017 28723550

[B177] Thangavel R. Bhagavan S. M. Ramaswamy S. B. Surpur S. Govindarajan R. Kempuraj D. (2017). Co-expression of glia maturation factor and apolipoprotein E4 in Alzheimer’s disease brain. *J. Alzheimer’s Dis.* 61 553–560. 10.3233/JAD-170777 29172001 PMC5770144

[B178] Thion M. S. Low D. Silvin A. Chen J. Grisel P. Schulte-Schrepping J. (2018). Microbiome influences prenatal and adult microglia in a sex-specific manner. *Cell* 172 500–516.e16. 10.1016/j.cell.2017.11.042 29275859 PMC5786503

[B179] Tiraboschi P. Hansen L. A. Masliah E. Alford M. Thal L. J. Corey-Bloom J. (2004). Impact of APOE genotype on neuropathologic and neurochemical markers of Alzheimer disease. *Neurology* 62 1977–1983. 10.1212/01.WNL.0000128091.92139.0F 15184600

[B180] Tong B. C.-K. Wu A. J. Li M. Cheung K.-H. (2018). Calcium signaling in Alzheimer’s disease & therapies. *Biochim. Biophys. Acta Mol. Cell Res.* 1865 1745–1760. 10.1016/j.bbamcr.2018.07.018 30059692

[B181] Trotta T. Panaro M. A. Cianciulli A. Mori G. Di Benedetto A. Porro C. (2018). Microglia-derived extracellular vesicles in Alzheimer’s disease: a double-edged sword. *Biochem. Pharmacol.* 148 184–192. 10.1016/j.bcp.2017.12.020 29305855

[B182] Trouche S. G. Maurice T. Rouland S. Verdier J.-M. Mestre-Francés N. (2010). The three-panel runway maze adapted to *Microcebus murinus* reveals age-related differences in memory and perseverance performances. *Neurobiol. Learn. Mem.* 94 100–106. 10.1016/j.nlm.2010.04.006 20403446 PMC2881169

[B183] Ulrich J. D. Finn M. Wang Y. Shen A. Mahan T. E. Jiang H. (2014). Altered microglial response to Aβ plaques in APPPS1-21 mice heterozygous for TREM2. *Mol. Neurodegener* 9:20. 10.1186/1750-1326-9-20 24893973 PMC4049806

[B184] Varma R. Chai Y. Troncoso J. Gu J. Xing H. Stojilkovic S. S. (2009). Amyloid-β induces a caspase-mediated cleavage of P2X4 to promote purinotoxicity. *Neuromolecular Med.* 11 63–75. 10.1007/s12017-009-8073-2 19562525 PMC2735730

[B185] Veerhuis R. Nielsen H. M. Tenner A. J. (2011). Complement in the brain. *Mol. Immunol.* 48 1592–1603. 10.1016/j.molimm.2011.04.003 21546088 PMC3142281

[B186] Verdonk F. Roux P. Flamant P. Fiette L. Bozza F. A. Simard S. (2016). Phenotypic clustering: a novel method for microglial morphology analysis. *J. Neuroinflammation* 13:153. 10.1186/S12974-016-0614-7 27317566 PMC4912769

[B187] Verheijen J. Sleegers K. (2018). Understanding Alzheimer disease at the interface between genetics and transcriptomics. *Trends Genet.* 34 434–447. 10.1016/j.tig.2018.02.007 29573818

[B188] Verkhratsky A. Zorec R. Rodríguez J. J. Parpura V. (2016). Astroglia dynamics in ageing and Alzheimer’s disease. *Curr. Opin. Pharmacol.* 26 74–79. 10.1016/j.coph.2015.09.011 26515274

[B189] Villa A. Gelosa P. Castiglioni L. Cimino M. Rizzi N. Pepe G. (2018). Sex-specific features of microglia from adult mice. *Cell Rep.* 23 3501–3511. 10.1016/j.celrep.2018.05.048 29924994 PMC6024879

[B190] Villegas-Llerena C. Phillips A. Reitboeck P. G. Hardy J. Pocock J. M. (2016). Microglial genes regulating neuroinflammation in the progression of Alzheimer’s disease. *Curr. Opin. Neurobiol.* 36 74–81. 10.1016/j.conb.2015.10.004 26517285

[B191] Viña J. Lloret A. (2010). Why women have more Alzheimer’s disease than men: gender and mitochondrial toxicity of amyloid-β peptide. *J. Alzheimer’s Dis.* 20 S527–S533. 10.3233/JAD-2010-100501 20442496

[B192] Vitek M. P. Brown C. M. Colton C. A. (2009). APOE genotype-specific differences in the innate immune response. *Neurobiol. Aging* 30 1350–1360. 10.1016/j.neurobiolaging.2007.11.014 18155324 PMC2782461

[B193] Wagner D.-C. Riegelsberger U. M. Michalk S. Härtig W. Kranz A. Boltze J. (2011). Cleaved caspase-3 expression after experimental stroke exhibits different phenotypes and is predominantly non-apoptotic. *Brain Res.* 1381 237–242. 10.1016/j.brainres.2011.01.041 21256117

[B194] Wagner D.-C. Scheibe J. Glocke I. Weise G. Deten A. Boltze J. (2013). Object-based analysis of astroglial reaction and astrocyte subtype morphology after ischemic brain injury. *Acta Neurobiol. Exp.* 73 79–87. 23595285 10.55782/ane-2013-1923

[B195] Wang J. Tanila H. Puoliväli J. Kadish I. van Groen T. (2003). Gender differences in the amount and deposition of amyloidβ in APPswe and PS1 double transgenic mice. *Neurobiol. Dis.* 14 318–327. 10.1016/J.NBD.2003.08.009 14678749

[B196] Wang Y. Cella M. Mallinson K. Ulrich J. D. Young K. L. Robinette M. L. (2015). TREM2 lipid sensing sustains the microglial response in an Alzheimer’s disease model. *Cell* 160 1061–1071. 10.1016/j.cell.2015.01.049 25728668 PMC4477963

[B197] Weinhard L. Neniskyte U. Vadisiute A. di Bartolomei G. Aygün N. Riviere L. (2018). Sexual dimorphism of microglia and synapses during mouse postnatal development. *Dev. Neurobiol.* 78 618–626. 10.1002/dneu.22568 29239126 PMC6001780

[B198] Wendt S. Maricos M. Vana N. Meyer N. Guneykaya D. Semtner M. (2017). Changes in phagocytosis and potassium channel activity in microglia of 5xFAD mice indicate alterations in purinergic signaling in a mouse model of Alzheimer’s disease. *Neurobiol. Aging* 58 41–53. 10.1016/j.neurobiolaging.2017.05.027 28697378

[B199] Wolf S. A. Boddeke H. W. G. M. Kettenmann H. (2017). Microglia in physiology and disease. *Annu. Rev. Physiol.* 79 619–643. 10.1146/annurev-physiol-022516-034406 27959620

[B200] Wyss-Coray T. Yan F. Lin A. H.-T. Lambris J. D. Alexander J. J. Quigg R. J. (2002). Prominent neurodegeneration and increased plaque formation in complement-inhibited Alzheimer’s mice. *Proc. Natl. Acad. Sci. U.S.A.* 99 10837–10842. 10.1073/pnas.162350199 12119423 PMC125059

[B201] Yang J.-T. Wang Z.-J. Cai H.-Y. Yuan L. Hu M.-M. Wu M.-N. (2018). Sex differences in neuropathology and cognitive behavior in APP/PS1/tau triple-transgenic mouse model of Alzheimer’s disease. *Neurosci. Bull.* 34 736–746. 10.1007/s12264-018-0268-9 30099679 PMC6129237

[B202] Yanguas-Casás N. Crespo-Castrillo A. de Ceballos M. L. Chowen J. A. Azcoitia I. Arevalo M. A. (2018). Sex differences in the phagocytic and migratory activity of microglia and their impairment by palmitic acid. *Glia* 66 522–537. 10.1002/glia.23263 29139169

[B203] Zhang W. Yan Z. Gao J. Sun L. Huang X. Liu Z. (2014). Role and mechanism of microglial activation in iron-induced selective and progressive dopaminergic neurodegeneration. *Mol. Neurobiol.* 49 1153–1165. 10.1007/s12035-013-8586-4 24277523 PMC4878835

[B204] Zhao W. Dumanis S. B. Tamboli I. Y. Rodriguez G. A. Jo LaDu M. Moussa C. E. H. (2014). Human APOE genotype affects intraneuronal A 1-42 accumulation in a lentiviral gene transfer model. *Hum. Mol. Genet.* 23 1365–1375. 10.1093/hmg/ddt525 24154541 PMC3919009

[B205] Zhao Y. Wu X. An Z. Huang T. Y. Xu H. Li X. (2018). TREM2 Is a receptor for β-amyloid that mediates microglial function. *Neuron* 97 1023–1031.e7. 10.1016/j.neuron.2018.01.031 29518356 PMC5889092

[B206] Zhong L. Wang Z. Wang D. Wang Z. Martens Y. A. Wu L. (2018). Amyloid-beta modulates microglial responses by binding to the triggering receptor expressed on myeloid cells 2 (TREM2). *Mol. Neurodegener.* 13:15. 10.1016/j.neuron.2016.05.003 29587871 PMC5870375

[B207] Zhou C.-N. Chao F.-L. Zhang Y. Jiang L. Zhang L. Luo Y.-M. (2018). Sex differences in the white matter and myelinated fibers of APP/PS1 mice and the effects of running exercise on the sex differences of AD mice. *Front. Aging Neurosci.* 10:243. 10.3389/fnagi.2018.00243 30174598 PMC6107833

[B208] Zhu Y. Nwabuisi-Heath E. Dumanis S. B. Tai L. M. Yu C. Rebeck G. W. (2012). APOE genotype alters glial activation and loss of synaptic markers in mice. *Glia* 60 559–569. 10.1002/glia.22289 22228589 PMC3276698

